# Uhrf1‐Mediated PKM2 Degradation via Ubiquitination Alleviates Inflammation and Pyroptosis in Inflammatory Bowel Disease

**DOI:** 10.1002/advs.77403

**Published:** 2026-08-25

**Authors:** Juan Zhang, Xin‐Rong Xu, Hui‐Lin Zhang, Qi‐Meng Zhu, Xin‐Yuan Li, Xi‐Peng Shang, Jing‐Hui Du, Christophe Morisseau, Feng Qiu, Cheng‐Peng Sun

**Affiliations:** ^1^ School of Chinese Materia Medica School of Medical Technology Tianjin Key Laboratory of Therapeutic Substance of Traditional Chinese Medicine Tianjin University of Traditional Chinese Medicine Tianjin People's Republic of China; ^2^ First Teaching Hospital of Tianjin University of Traditional Chinese Medicine Tianjin People's Republic of China; ^3^ Department of Entomology and Nematology UC Davis Comprehensive Cancer Center University of California Davis California USA

**Keywords:** eupatolide, Inflammatory bowel disease, Interaction, PKM2, Uhrf1

## Abstract

Inflammatory bowel disease (IBD) is characterized by chronic relapsing intestinal inflammation and closely associated with persistent inflammatory responses and impairment of the mucosal barrier integrity. In this work, we demonstrated that Th17/Treg immune imbalance drove inflammation and pyroptosis in IBD, as revealed by single‐cell RNA sequencing, and pyruvate kinase M2 (PKM2) and ubiquitin‐like with PHD and RING finger domains 1‌ (Uhrf1) displayed a negative correlation in IBD clinic samples. Target fishing analysis revealed that eupatolide (EPT) covalently bound to C165 of PKM2 with a dissociation constant (*K*_d_) of 106 nM, corroborated by follow‐up chemical biology assays. We gained a deeper understanding of the mechanistic by which EPT interfered PKM2 function, specifically by promoting its interaction with Uhrf1, enhancing the K48‐linked ubiquitylation and degradation of PKM2 to block its nuclear translocation. Critically, PKM2 silencing mitigated dextran sodium sulfate (DSS)‐driven inflammation and pyroptosis across in vitro and in vivo models, with EPT showing no further efficacy upon *PKM2* knockdown in IBD mice. This work uncovered Uhrf1‐dependent PKM2 ubiquitination/degradation as a previously unrecognized therapeutic axis for IBD, while positioning EPT as a molecular glue capable of targeting the Uhrf1–PKM2 complex.

## Graphical abstract

Uhrf1‐mediated PKM2 ubiquitination and degradation repressed the nuclear translocation of PKM2, and EPT served as a molecular glue capable of targeting the Uhrf1–PKM2 complex to alleviate the IBD course, suggesting that the Uhrf1–PKM2 axis was a previously unrecognized strategy for treating IBD.

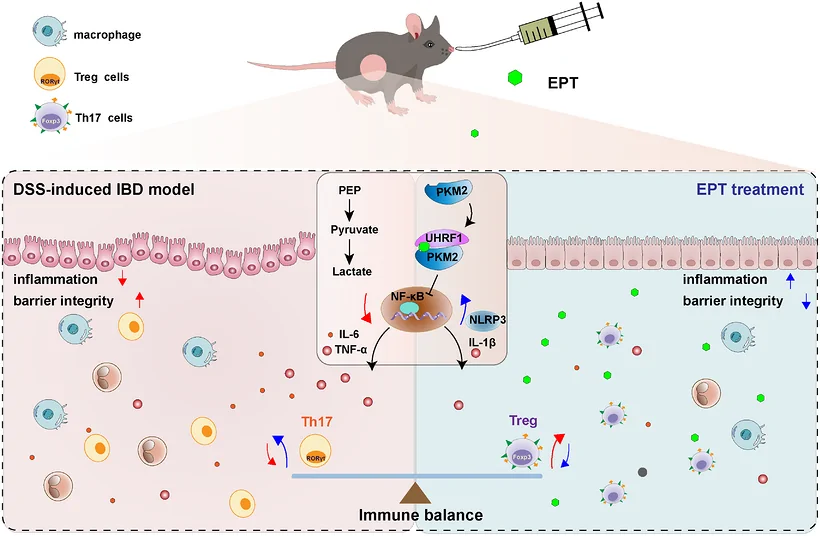

## 1 Introduction

Inflammatory bowel disease (IBD), an immune‐mediated chronic inflammatory disorder of the gut, includes Crohn's disease and ulcerative colitis, both exhibiting global incidence patterns and affecting over 5 million people globally [1, 2, 3]. Incidence persists in the Western world, though now stable at 0.25%–0.4% [4]. Consequently, estimates indicate 1% of Western populations could be affected by 2028, demanding significant adaptations in healthcare infrastructure [5, 6]. IBD patients frequently confront a constellation of risk factors that contribute to disease exacerbation or complications over time [7, 8]. These etiological elements induce structural and functional impairments in the colon, resulting in pathophysiological manifestations, such as impaired colonic motility and anorectal malfunction [9]. Despite extensive genome‐wide association studies uncovering a multitude of IBD‐associated susceptibility loci, the precise pathogenic mechanisms remain elusive, thereby complicating therapeutic development targeting molecular pathways [10]. While small‐molecule drugs and biologics are currently available for treating IBD [11], the disease cannot be cured, and no single treatment regimen is effective for all patients. Therefore, there remains an urgent need to explore novel therapeutic strategies for IBD.

Pyruvate kinase M2 (PKM2), a crucial isoform of pyruvate kinase (PK), serves as a key enzyme in glucose metabolism and is involved in the regulation of cellular energy metabolism [12, 13]. In glycolysis, PKM2 catalyzes the conversion of phosphoenolpyruvate (PEP) to pyruvate, acting as a pivotal rate‐limiting enzyme in the glycolytic pathway that modulates the flux of glucose metabolism and energy generation [13]. PKM2 exists in two interconvertible conformations, namely dimer and tetramer. The tetramer exhibits high glycolytic activity, thereby facilitating the progression of aerobic glycolysis [14, 15]. In contrast, the dimer form possesses low enzymatic activity and primarily translocates into the nucleus to interact with multiple transcription factors [16, 17]. Furthermore, PKM2 is capable of regulating the balance between T helper 17 (Th17) cells and regulatory T (Treg) cells: it promotes the differentiation and proliferation of Th17 cells while inhibiting the function of Treg cells, thereby orchestrating the direction of immune responses [18]. Meanwhile, PKM2 can modulate the M1/M2 polarization of macrophages, either promoting the activation of M1‐type macrophages to amplify inflammatory responses or inducing M2‐type polarization to participate in tissue repair [19]. Abnormal expression or conformational imbalance of PKM2 is associated with a variety of diseases. Particularly in IBD, its aberrant activation exacerbates intestinal inflammatory responses, impairs immune homeostasis, and contributes to the occurrence and progression of the disease [20]. However, the underlying mechanism of PKM2‐mediated IBD remains limited.

Natural products, evolved under selective pressure to engage biological macromolecular targets, constitute an indispensable reservoir for drug discovery [21, 22, 23, 24]. This significance is underscored by the fact that over 50% of drugs approved by the US Food and Drug Administration derive directly from natural sources or their derivatives [25]. Recent advances corroborate the therapeutic efficacy of specific natural products, such as 1‐*O*‐acetyl‐4*R*,6*S*‐britannilactone, kurarinone, wedelolactone, and alisol B, in preclinical studies [26, 27, 28, 29, 30, 31]. Aucklandiae Radix is the dried root of *Aucklandia lappa* Decne., and documented in the Chinese Pharmacopoeia for its ability to strengthen intestinal function and alleviate diarrhea [7]. It is widely used for managing various digestive disorders in the clinic [32]. Our prior investigation established that Aucklandiae Radix ameliorated IBD via dual‐action immunomodulation: suppressing intestinal macrophage/neutrophil infiltration while restoring Th17/Treg homeostasis [7]. In this work, we employed single‐cell RNA sequencing (scRNA‐seq) analysis to reveal that Th17/Treg immune deficiency played a critical role in driving inflammatory response and pyroptosis in IBD. Through screening our in‐house Aucklandiae Radix library, we identified eupatolide (EPT) as a bioactive small molecule exhibiting potent immunoregulatory, anti‐inflammatory, and anti‐pyroptotic activities. Using EPT as a small‐molecule probe, our findings suggested that PKM2 was associated with IBD and overexpressed in IBD clinical samples. Mechanistically, ubiquitin‐like with PHD and RING finger domains 1‌ (Uhrf1) promoted Lys48 (K48)‐linked ubiquitylation and degradation of PKM2 to block its nuclear translocation, exerting the anti‐IBD effect for alleviating inflammation and pyroptosis. Critically, PKM2 silencing mitigated dextran sodium sulfate (DSS)‐driven inflammation and pyroptosis across in vitro and in vivo models, with EPT showing no further efficacy upon *PKM2* knockdown in IBD mice.

## 2 Results

### 2.1 Imbalance of Th17/Treg Immune Cells, NF‐κB Signal Transduction, and the Pyruvate Metabolic Process Involved the Occurrence and Development of IBD

In order to investigate the development curve of IBD, we obtained scRNA‐seq data of 17 donors for healthy and IBD from the NCBI database, and analyzed the characteristics and proportional changes of different cell clusters using bioinformatics methods. After quality control, filtering, dimensionality reduction, and clustering, the cell distribution map was observed in Figure 1A, and 9 kinds of cell clusters (Figure 1B) were divided based on the characteristic genes (Figure 1C). The proportions of B, endothelial, macrophage, smooth muscle, and T cells in the IBD group were significantly elevated compared with the healthy group (Figure 1D), and those of epithelial cells and plasma cells were decreased (Figure 1D). Further analysis of T cell subsets revealed that an imbalance in the ratio of Th17 cells to Treg cells was observed in IBD patients (Figure 1E). Leveraging an integrated scRNA‐seq dataset, we systematically characterized the immune landscape heterogeneity between healthy controls and IBD patients. Transcriptomic profiling revealed that both RELA (p65 subunit) and NLR family pyrin domain containing 3 (NLRP3) inflammasome components exhibited statistically significant overexpression in IBD specimens relative to healthy tissues (Figure 1H,I). Subsequent functional enrichment analysis demonstrated prominent activation signatures in pyruvate metabolism and canonical NF‐κB cascade pathways within the IBD cohort (Figure 1J,K). These integrative analyses provided compelling evidence implicating dysregulated NF‐κB/NLRP3 axis activity and imbalance of Th17/Treg immune cells as a critical driver of the inflammation pathogenesis in IBD, operating synergistically with metabolic reprogramming mechanisms characteristic of active disease states.

**FIGURE 1:**
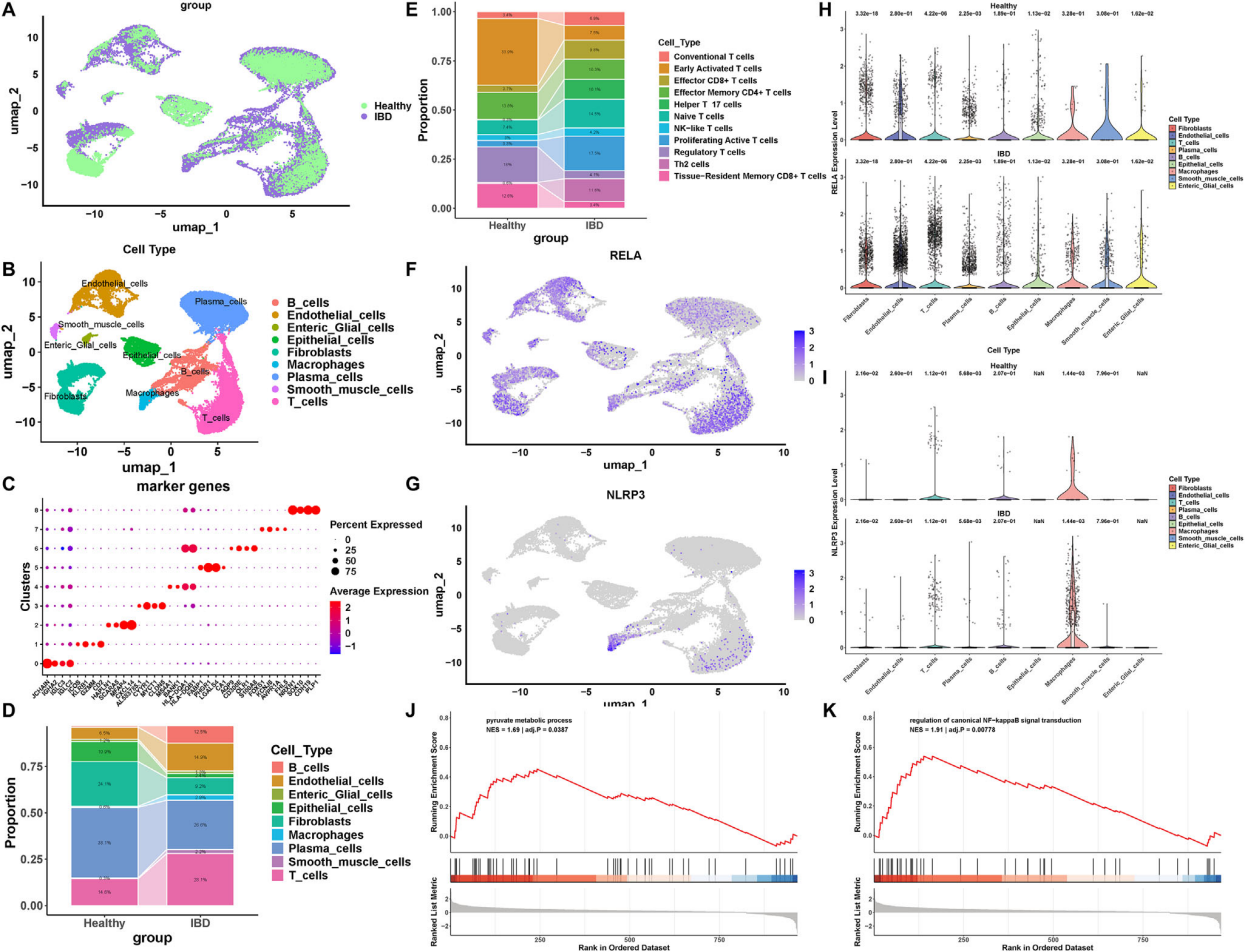
Imbalance of Th17/Treg immune cells, NF‐κB signal transduction, and pyruvate metabolic process were associated with the occurrence and development of IBD. (A) UMAP visualization of cells from healthy and IBD; (B) Cell type distribution in UMAP after manual annotation of each cluster based on known marker genes; (C) Top4 characteristic genes with the highest expression levels within each cell population; (D) Stacked bar chart showing the relative abundance of each cell type in healthy and IBD; (E) Stacked bar chart of the proportion of each subpopulation after further subdivision of T cell population; (F, G) UMAP feature maps showing the expression distribution of RELA (encoding p65) and NLRP3; (H, I) Expression levels of RELA and NLRP3 across different cell types; (J, K) GSEA results focused on the pyruvate metabolism and NF‐κB pathways.

### 2.2 EPT Alleviated the Course of DSS‐Mediated IBD in a Mouse Model

Based on the characteristics of IBD, we focused on inflammatory response and immunity to screen the library of our natural products. It was notable that EPT (Figure 2A), isolated from Aucklandiae Radix that was a traditional Chinese medicine recorded in the Chinese Pharmacopoeia, displayed the potential for treating IBD (Figure 2B). As shown in Figure 2C, EPT administration dose‐dependently reversed the decrease in body weight and the increase in scores for diarrhea, rectal bleeding, and disease activity index (DAI) in DSS‐mediated IBD mice (Figure 2C and Figure S1). Following EPT administration, IBD mice exhibited dose‐dependent colonic elongation (Figure 2D) with significant attenuation of gastrointestinal hemorrhage and concurrent restoration of vascular integrity (Figure 2E). H&E and occludin immunostaining revealed that EPT administration dose‐dependently mitigated inflammatory cell infiltration and preserved intestinal crypt architecture in DSS‐induced IBD mice (Figure 2F). This therapeutic effect was substantiated by upregulated occludin expression (Figure 2F and Figure S2), which fortified intestinal barrier integrity and subsequently downregulated inflammatory cytokines, including IL‐6, IL‐1α, IL‐1β, CXCL10, and CCL5 (Figure 2G and Figure S3), highlighting EPT's therapeutic potential for IBD management.

**FIGURE 2:**
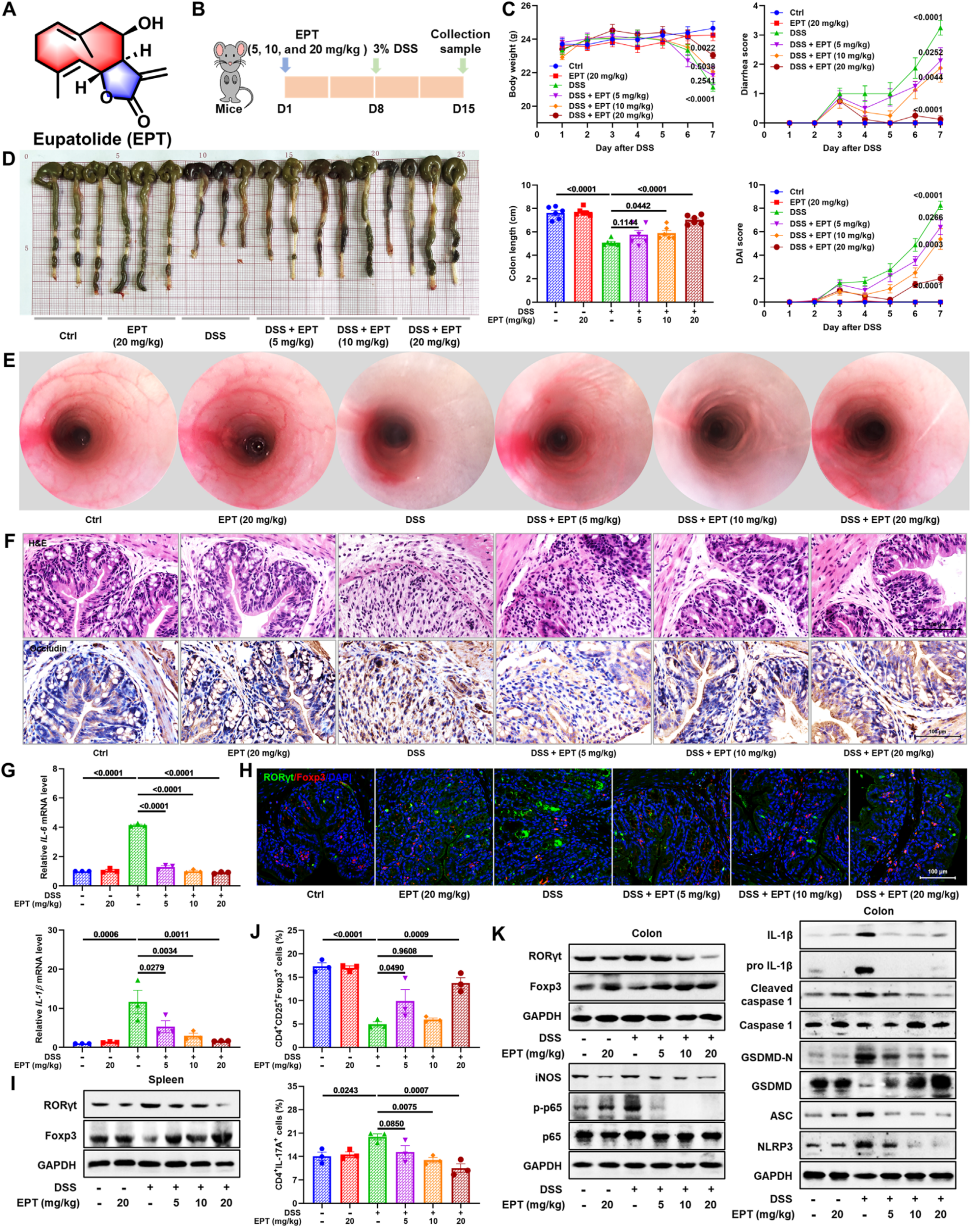
EPT regulated the balance of Th17/Treg immune cells and inflammation to alleviate the course of DSS‐mediated IBD in a mouse model. (A) Structure of EPT; (B) Timeline for the DSS‐mediated IBD experiment; (C) Body weight, diarrhea score, and DAI score (*n* = 8); (D) Gross colon morphology and length (*n* = 6); (E) Colonoscopy plots; (F) H&E and occludin staining plots; (G) Effects of EPT toward mRNA levels of *IL‐6* and *IL‐1β* (*n* = 3); (H) RORγt/Foxp3 staining plots; (I) Effects of EPT toward expressions for RORγt and Foxp3 in spleen (*n* = 3); (J) Flow cytometry results for Th17 (CD4^+^IL‐17A^+^) and Treg (CD4^+^CD25^+^Foxp3^+^) cells (*n* = 3) after defining the gate of CD4^+^ T cells by FITC‐CD4 anti‐body; (K) EPT regulated expressions of RORγt and Foxp3, and suppressed NF‐κB and NLRP3 pathways involved in iNOS, p‐p65, p65, IL‐1β, pro IL‐1β, cleaved caspase 1, caspase 1, GSDMD‐N, GSDMD, ASC, and NLRP3.

### 2.3 EPT Restored the Balance of Th17/Treg Immune Cells to Alleviate Intestinal Inflammation Through NF‐κB and NLRP3 Pathways In Vivo

scRNA‐seq analysis comparing healthy controls and IBD patients identified dysregulation in Th17/Treg cell balance, NF‐κB, and NLRP3 pathways as key features of IBD pathogenesis. Consequently, we evaluated EPT's immunoregulatory potential, specifically its suppressive effects on the NF‐κB and NLRP3 inflammasome pathways. EPT administration significantly reduced CD4^+^IL‐17A^+^ Th17 cells while increasing CD4^+^CD25^+^Foxp3^+^ Tregs in DSS‐induced IBD mice (Figure 2J and Figure S4A), consistent with suppressed *RORc* (RORγt) and elevated *Foxp3* mRNA/protein expressions in colonic and splenic tissues (Figure 2H,I,K and Figures S4B,S5,S6). Concomitantly, EPT inhibited NF‐κB and NLRP3 inflammasome activation, evidenced by decreased iNOS, p‐p65, IL‐1β, pro IL‐1β, cleaved caspase‐1, GSDMD‐N, ASC, and NLRP3 levels (Figure 2K and Figure S7), alongside reduced COX‐2, ASC, and NLRP3 immunoreactivity in colonic sections (Figure S8). These data demonstrated that EPT exerted immunomodulatory and anti‐inflammatory effects via disruption of NF‐κB‐driven inflammation and NLRP3‐mediated pyroptosis.

### 2.4 EPT Possessed Anti‐Inflammatory, Anti‐Pyroptosis, and Mitochondrial Protective Effects In Vitro

Based on the cytotoxicity result, EPT exhibited no significant toxic effects under 2 µM (Figure S9). However, within this non‐toxic range, EPT dose‐dependently inhibited the LPS‐induced release of key inflammatory factors, such as nitric oxide (NO), IL‐6, and TNF‐α (Figure 3A) in RAW264.7 cells. Consistent with these findings, EPT significantly suppressed mRNA expressions of pro‐inflammatory genes (e.g., *iNOS*, *COX‐2*, *IL‐1α*, *IL‐6*, and *CCL5*) in LPS‐stimulated inflammatory cells (Figure 3D and Figure S10). LPS exposure induced pronounced mitochondrial dysfunction, characterized by decreased expression of mitochondrial fusion proteins (e.g., Mfn1, Mfn2, and Opa1), increased expression of fission protein Fis1, and elevated release of mitochondrial DNA (mtDNA) (Figure 3G and Figures S10–S12). This dysfunction contributed to disrupted cellular metabolism, evidenced by increased calcium influx and lactic acid production (Figure 3B,C). Crucially, EPT treatment effectively reversed these LPS‐induced alterations. It restored expression levels of critical mitochondrial dynamics proteins (e.g., Mfn1, Mfn2, Opa1, and Fis1), normalized metabolic markers (e.g., LDHA, HK2, and GLUT1), and suppressed the inflammatory signaling molecule p‐p65 alongside iNOS and COX‐2 (Figure 3E,G,H). Immunofluorescence staining for COX‐2 and p65 further corroborated the inhibitory effect of EPT on inflammatory pathways (Figure 3H).

**FIGURE 3:**
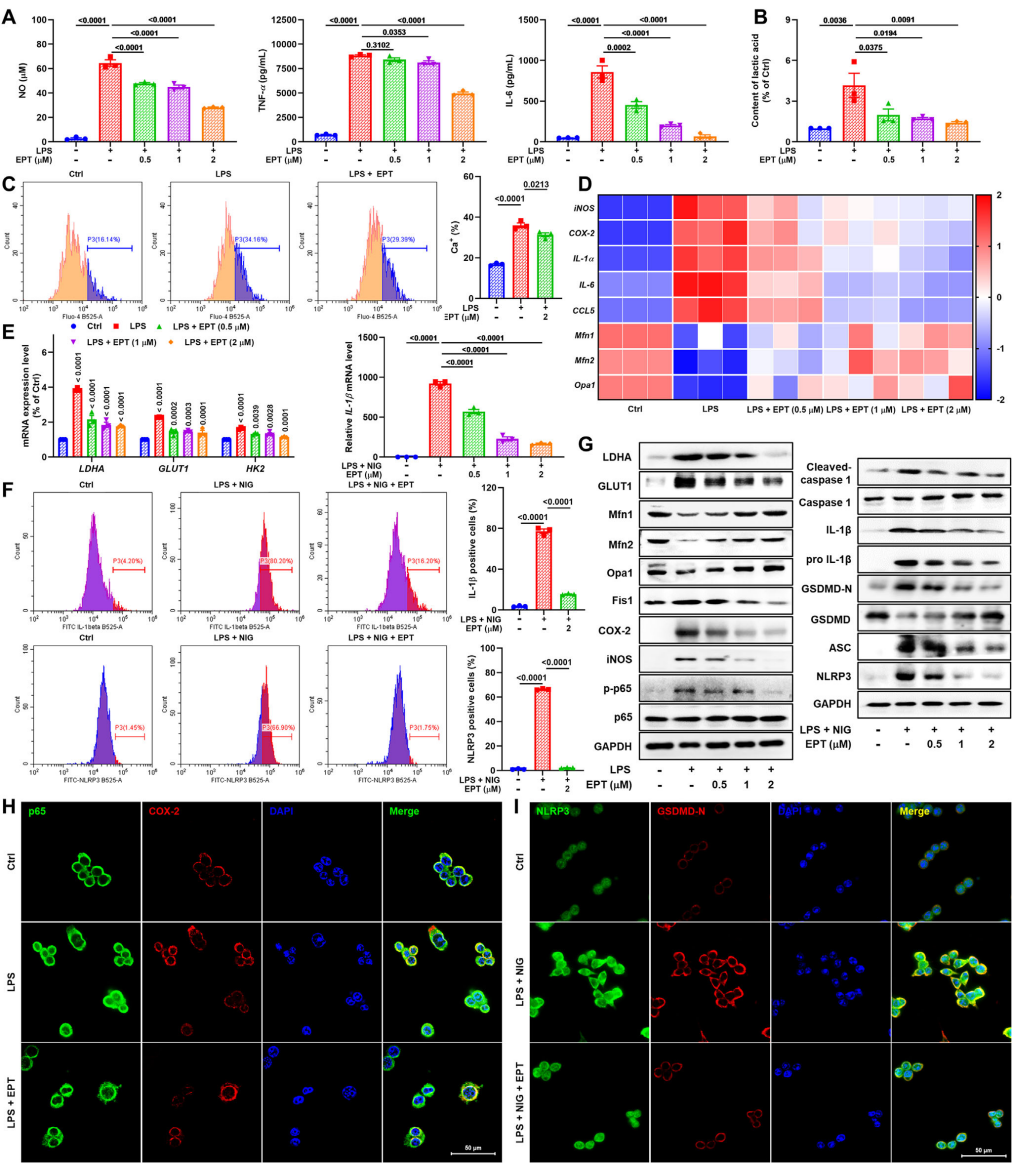
EPT suppressed NF‐κB‐mediated inflammation and NLRP3‐mediated pyroptosis in vitro. (A) Effects of EPT toward inflammatory factors NO, TNF‐α, and IL‐6 (*n* = 3); (B) Effect of EPT toward lactic acid (*n* = 3); (C) Flow cytometry results of calcium ion (Ca^+^, Fluo‐4, *n* = 3); (D) Heatmap of mRNA levels for *iNOS*, *COX‐2*, *IL‐1α*, *IL‐6*, *CCL5*, *Mfn1*, *Mfn2*, and *Opa1* (*n* = 3); (E) Effects of EPT toward mRNA levels for *LDHA, HK2, GLUT1*, and *IL‐1β* (*n* = 3); (F) Flow cytometry results of *IL‐1β* and *NLRP3* (*n* = 3); (G) Effects of EPT toward expressions of proteins involved in mitochondrion, lactic acid metabolism, NF‐κB, and NLRP3 pathways; (H, I) p65/COX‐2 and NLRP3/GSDMD‐N immunofluorescence staining plots.

Flow cytometry analyses revealed that EPT significantly attenuated the increase in IL‐1β^+^ or NLRP3^+^ cell populations (Figure 3F) and the loss of mitochondrial membrane potential observed in LPS/NIG‐treated cells (Figure S13). LPS/NIG co‐treatment promoted caspase‐1 activation, pro IL‐1β maturation (IL‐1β), and GSDMD cleavage, alongside upregulated expression of pro IL‐1β, ASC, and NLRP3 (Figure 3G and Figure S14). These effects were reversed upon EPT administration. Mechanistically, immunofluorescence staining confirmed that EPT disrupted NLRP3 inflammasome assembly, as evidenced by reduced colocalization of NLRP3 (green) with GSDMD‐N (red, Figure 3I), thereby substantiating its inhibitory role in pyroptosis.

### 2.5 PKM2 was Identified as a Direct Target of EPT Through the Biotin Labeling and Fluorescence Imaging Technology

Next, the biotin labeling and fluorescence imaging technology were applied to discover the target of EPT (Figure 4A). EPT probe labeled with biotin (Bio‐EPT, Figure 4B) was synthesized and utilized in pull‐down assays, which revealed a prominent ∼58 kDa protein band. Subsequent mass spectrometry analysis (LC‐MS/MS) and immunoblotting using anti‐PKM2 antibody confirmed this band as PKM2 (Figure 4C). As shown in Figure 4D, EPT dose‐dependently stabilized PKM2 against proteolytic degradation (pronase) and thermal denaturation in DARTS and CETSA experiments (Figure S15). Consistent with these findings, concentration‐dependent thermal stabilization of PKM2 by EPT was further corroborated via CETSA, underscoring their direct interaction (Figure 4D and Figure S15). Confocal microscopy‐based colocalization studies visualized immediate binding between fluorescently labeled EPT and endogenous PKM2 in cellular compartments (Figure 4E). This interaction was quantitatively characterized by MST, revealing high‐affinity binding with an equilibrium dissociation constant (*K*_d_) of 106 nM (Figure 4F). PKM2 was related to oxidative phosphorylation (OXPHOS) and glycolysis, therefore, we further assayed effects of EPT on oxygen consumption rate (OCR) and proton extrusion rate‌ (PER) using the Seahorse instrument. As shown in Figure 4G, EPT reversed the decrease of LPS‐mediated basal respiration, proton leak, Max. respiration, and ATP production, while reducing levels of basal glycolysis, basal proton efflux rate, and compensatory glycolysis in LPS‐mediated RAW264.7 cells, suggesting that EPT regulated OXPHOS and glycolysis reprogramming. Collectively, these multi‐faceted approaches established PKM2 as a direct target of EPT.

**FIGURE 4:**
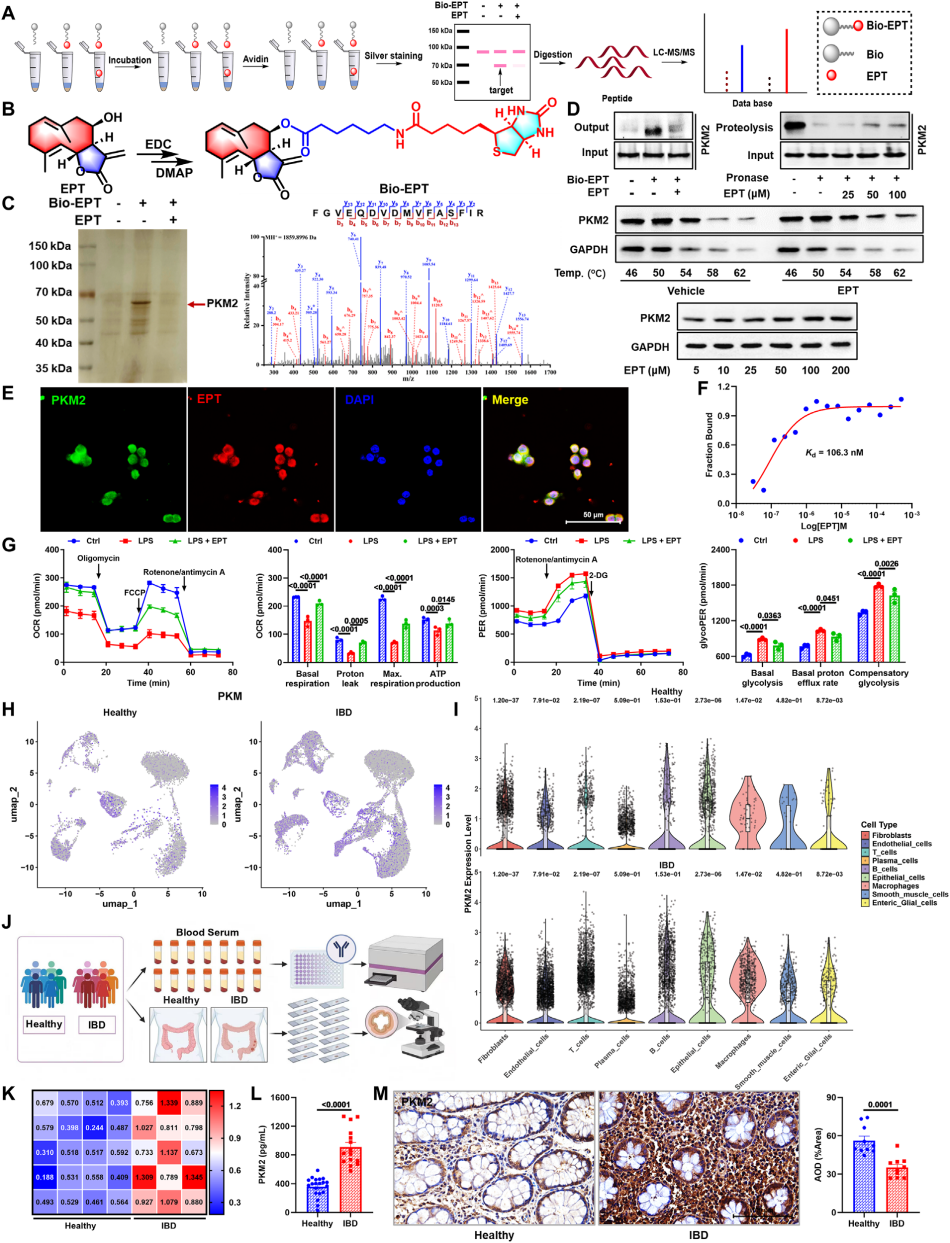
PKM2 was defined as the direct target of EPT. (A) Schematic diagram of fishing target technology; (B) The synthesis of the biotinylated probe Bio‐EPT; (C) Silver staining and LC‐MS/MS plots; (D) Pull down, DARTS, and CETSA (varied with temperature or EPT concentration) plots detected by Western blot; (E) Immunofluorescence co‐localization of EPT with PKM2; (F) MST plot of EPT with PKM2; (G) OXPHOS and glycolysis experimental results (*n* = 3); (H) UMAP feature maps showing the expression distribution of PKM2 in healthy and IBD groups analyzed by scRNA‐seq; (I) Expression level of PKM2 across different cell types; (J) Workflow schematic for analyzing contents of PKM2 and its expression in clinic samples; (K) OD value of PKM2 detected by its corresponding Elisa kit (*n* = 15–20); (L) PKM2 contents in bloods of healthy and IBD groups (*n* = 15–20); (M) PKM2 staining plots in intestinal tissues of healthy and IBD groups (*n* = 10).

### 2.6 PKM2 was Associated with the Progression of IBD

To explore the function of PKM2 in IBD, we analyzed scRNA‐seq data of healthy and IBD patients from the NCBI database and revealed a significant difference in PKM2 expression between the healthy and IBD groups. The distribution of PKM2 expression was shown in Figure 4H. Further quantitative analysis across distinct cell clusters demonstrated that PKM2 expression was significantly upregulated in multiple cell populations, including fibroblasts and T cells, in IBD samples (Figure 4I). We collected serum and intestinal tissue samples from healthy and IBD patients (Figure 4J), and found that the PKM2 concentration in serum for IBD patients was approximately two‐fold higher in the healthy control (Figure 4K,L). Consistent with this, our IHC analysis illustrated that PKM2 was overexpressed in intestinal tissues for IBD (Figure 4M).

Next, we engineered plasmid‐mediated PKM2‐specific siRNA for knockdown to elucidate the functional role of PKM2, which contributed to a decrease of mRNA and protein expression (Figure 5A). Conversely, the rescue of *PKM2* brought about overexpression of its corresponding mRNA and protein (Figure 5E). In *PKM2* knockdown cells, LPS‐induced increases in mRNA/protein levels of IL‐1β, pro IL‐1β, IL‐1α, IL‐6, LDHA, HK2, GLUT1, Drp1, iNOS, Mfn1, Mfn2, COX‐2, p‐p65, cleaved‐caspase 1, ASC, and NLRP3, and the decrease in OPA1 mRNA were reversed, along with suppression of NF‐κB and NLRP3 activation (Figure 5B–D and Figures S16,S17). Notably, protective effects of EPT were revoked in LPS or LPS plus NIG‐exposed *PKM2* knockdown cells (Figure 5B–D and Figures S16,S17). In contrast, *PKM2* overexpression worsened inflammation and pyroptosis triggered by LPS or LPS/NIG, and critically, eliminated EPT's ability to suppress these processes (Figure 5F–H and Figures S18, and S19). This confirmed that EPT required functional PKM2 to exert protection.

**FIGURE 5:**
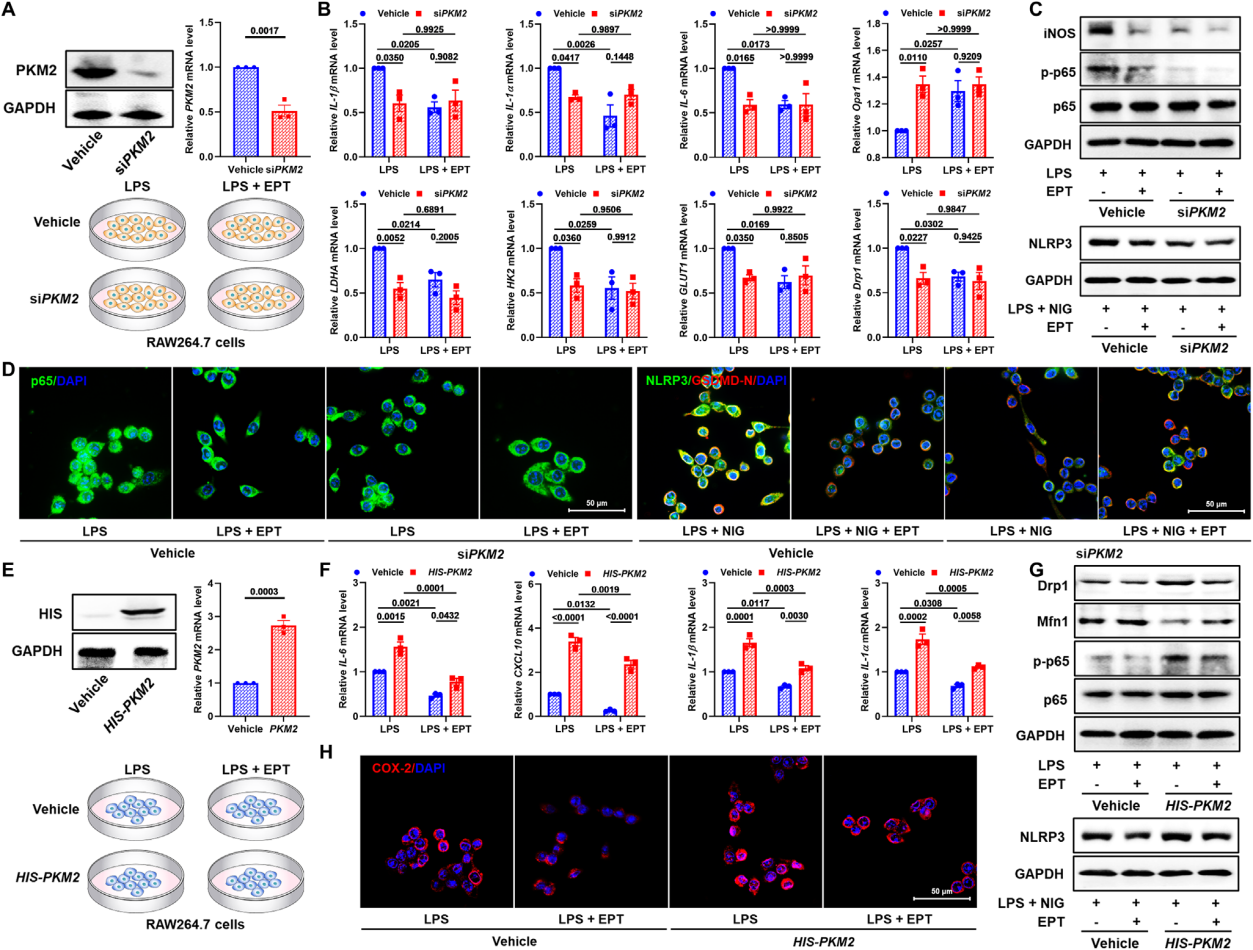
PKM2 knockdown or overexpression revoked protective effects of EPT in vitro. (A) *PKM2* knockdown verification (*n* = 3); (B) *PKM2* knockdown abolished effects of EPT toward mRNA levels of *IL‐1β*, *IL‐1α*, *IL‐6*, *Opa1*, *Drp1*, *LDHA*, *HK2*, and *GLUT1* (*n* = 3); (C) *PKM2* knockdown abolished effects of EPT toward expression of iNOS, p‐p65, p65, and NLRP3; (D) p65, NLRP3, and GSDMD‐N staining plots; (E) *PKM2* overexpression verification (*n* = 3); (F) *PKM2* overexpression weakened effects of EPT toward mRNA levels of *IL‐6*, *IL‐1α*, *CXCL10*, and *IL‐1β* (*n* = 3); (G) *PKM2* overexpression weakened effects of EPT toward expression of Drp1, Mfn1, p‐p65, p65, and NLRP3; (H) COX‐2 staining plots.

### 2.7 PKM2 C165 was the Covalent Binding Site of EPT

To determine critical amino acid residues in the EPT‐PKM2 interaction interface, a competition experiment employing iodoacetamide (IAA), a cysteine‐specific alkylating agent, was employed. Pre‐incubation with IAA reduced Bio‐EPT‐mediated PKM2 capture (Figure 6A), mirroring the effect observed when EPT was pre‐incubated alone, demonstrating EPT's covalent binding capacity to PKM2. After the incubation of EPT with recombinant human PKM2 (Figure 6B), LC‐MS/MS analysis demonstrated that EPT covalently bound to Cys165 (C165) and C474 of PKM2 because the molecular weights of the sequences for KNICKVVEVGSKIY and CKDPVQEAW were both increased by 248.1412 Da (Figure 6C). Next, we mutated C165 and C474 of PKM2 into Cys165Ala (C165A) and C474A, and found that the C165A mutation disrupted EPT‐PKM2 binding rather than the C474A mutation (Figure 6D), while diminishing EPT's protein‐stabilizing properties toward PKM2, as evidenced by both CETSA and DARTS assays (Figure 6D). The anti‐inflammatory efficacy of EPT was abolished in LPS‐stimulated cells expressing the C165A‐mutated PKM2 (Figure 6E). Notably, this mutation significantly reduced EPT's binding affinity to PKM2, with a markedly increased dissociation constant (*K*_d_ = 26.42 µM, Figure 6F). Consistently, molecular docking analysis further corroborated the covalent interaction between EPT and the C165 residue of PKM2 (Figure 6G). As shown in Figure 6H,I, C165A‐mutated PKM2 recue activated NF‐κB and NLRP3 pathways to promote the increasing of mRNA and protein levels for key genes (such as *IL‐6*, *IL‐1β*, and *CXCL10*) and proteins (such as iNOS, COX‐2, pro IL‐1β, and IL‐1β), while EPT treatment did not have extra effects in LPS‐ or LPS plus NIG‐mediated PKM2 knockdown cells after PKM2 C165A recue. These results collectively established C165 as the critical binding site for EPT's pharmacological action.

**FIGURE 6:**
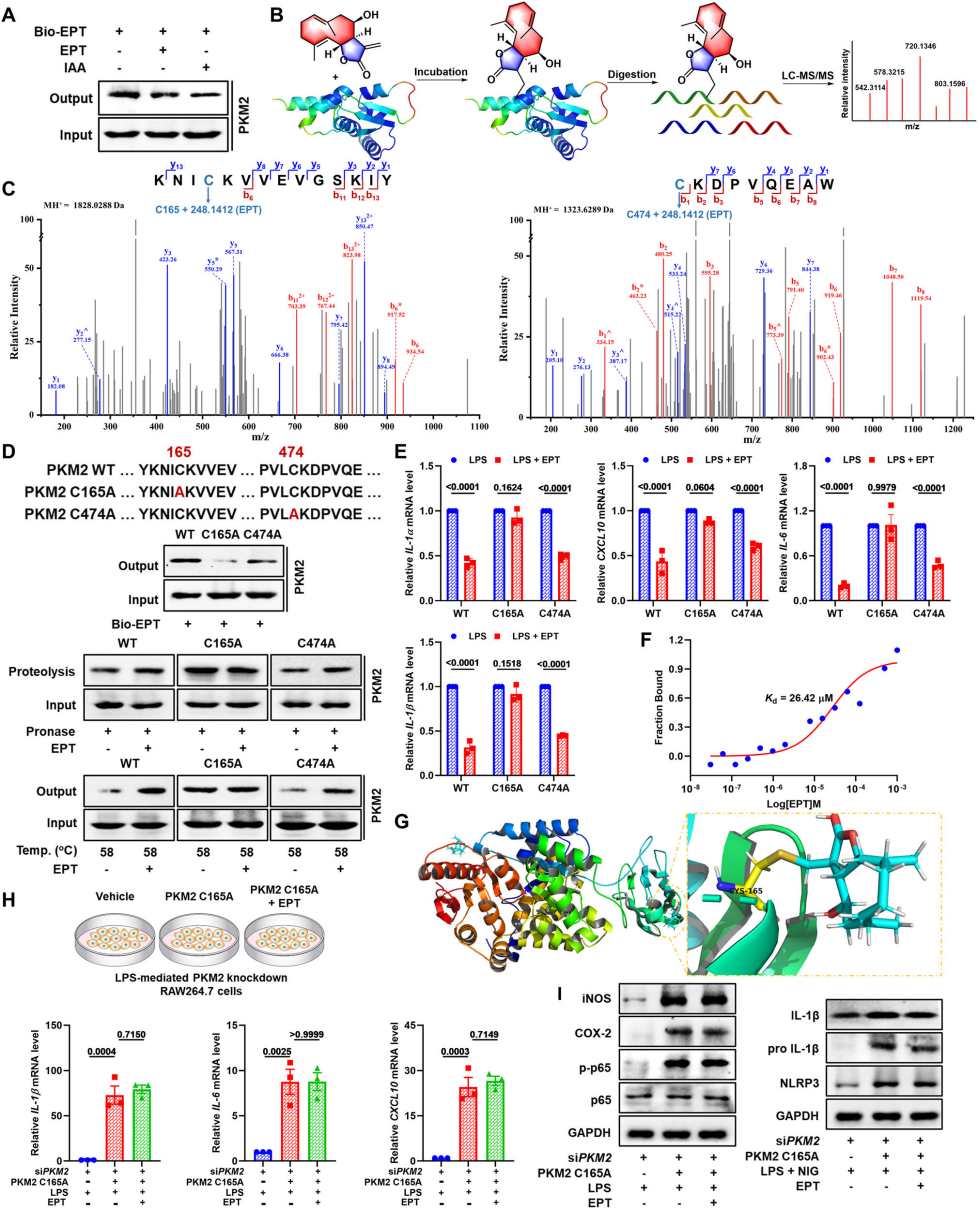
EPT specifically modified amino acid residue C165 of PKM2. (A) IAA disrupted the binding affinity between EPT and PKM2; (B) Schematic diagram of the covalently binding identification of EPT with PKM2; (C) LC‐MS/MS plots; (D) Cys165Ala (C165A) mutation weakened the binding of EPT with PKM2 rather than Cys474Ala (C474A) mutation; (E) C165A mutation abolished anti‐inflammatory effects of EPT rather than C474A mutation in vitro (*n* = 3); (F) MST plot of EPT with PKM2 C165A; (G) 3D interaction of EPT with PKM2; (H) PKM2 C165A recue enhanced mRNA levels of *IL‐1β*, *IL‐6*, and *CXCL10* in LPS‐mediated PKM2 knockdown cells (*n* = 3); (I) PKM2 C165A recue activated NF‐κB and NLRP3 pathways in LPS or LPS plus NIG‐mediated PKM2 knockdown cells.

### 2.8 Uhrf1‐Dependent K48‐Linked Ubiquitination Drove PKM2 Degradation

Beyond its canonical role in metabolizing PEP, PKM2 exhibited nuclear translocation to modulate *LDHA* and *GLUT1* transcription, exerting a pro‐inflammatory effect after LPS exposure. In follow‐up experiments, we observed that EPT attenuated the LPS‐induced upregulation of PKM2 and repressed its nuclear translocation (Figure 7A,B). Pretreatment with the proteasome inhibitor MG132, but not the protein synthesis inhibitor CHX, abolished the inhibitory effect of EPT (Figure 7C), demonstrating that PKM2 degradation occurred via the proteasomal pathway. In order to investigate the underlying mechanism, we utilized the ascorbate peroxidase 2 (APEX2) proximity labeling technique to search for the key interaction protein of PKM2 (Figure 7D). Analysis of the volcano plot demonstrated 17 differentially proteins, including adhesion G protein‐coupled receptor E1 (Adgre1), Uhrf1, and cytochrome c oxidase subunit 7A2 (Cox7a2), as visualized in Figure 7E,F. Gene ontology analysis identified Uhrf1 as an E3 ubiquitin‐protein ligase, suggesting it might serve as a critical interactor of PKM2 involved in mediating its degradation within this protein set. Uhrf1 staining plots indicated that it was downregulated (Figure 7G,H), and it was negatively correlated with PKM2 (Figure 7I) after analyzing healthy and IBD patients. Co‐IP assays in HEK293T cells expressing GST‐tagged PKM2 and HA‐tagged Uhrf1 demonstrated their interaction, and EPT enhanced their interaction, particularly under LPS stimulation (Figure 7J,K and Figure S20), which was supported through the MST experiment (*K*_d_, 0.64 µM for PKM2 with Uhrf1; *K*_d_, 0.16 µM for PKM2 with Uhrf1 in the presence of EPT, Figure 7M). However, the PKM2 C165A mutation abolished this EPT‐mediated promotion, as evidenced by Figure 7L.

**FIGURE 7:**
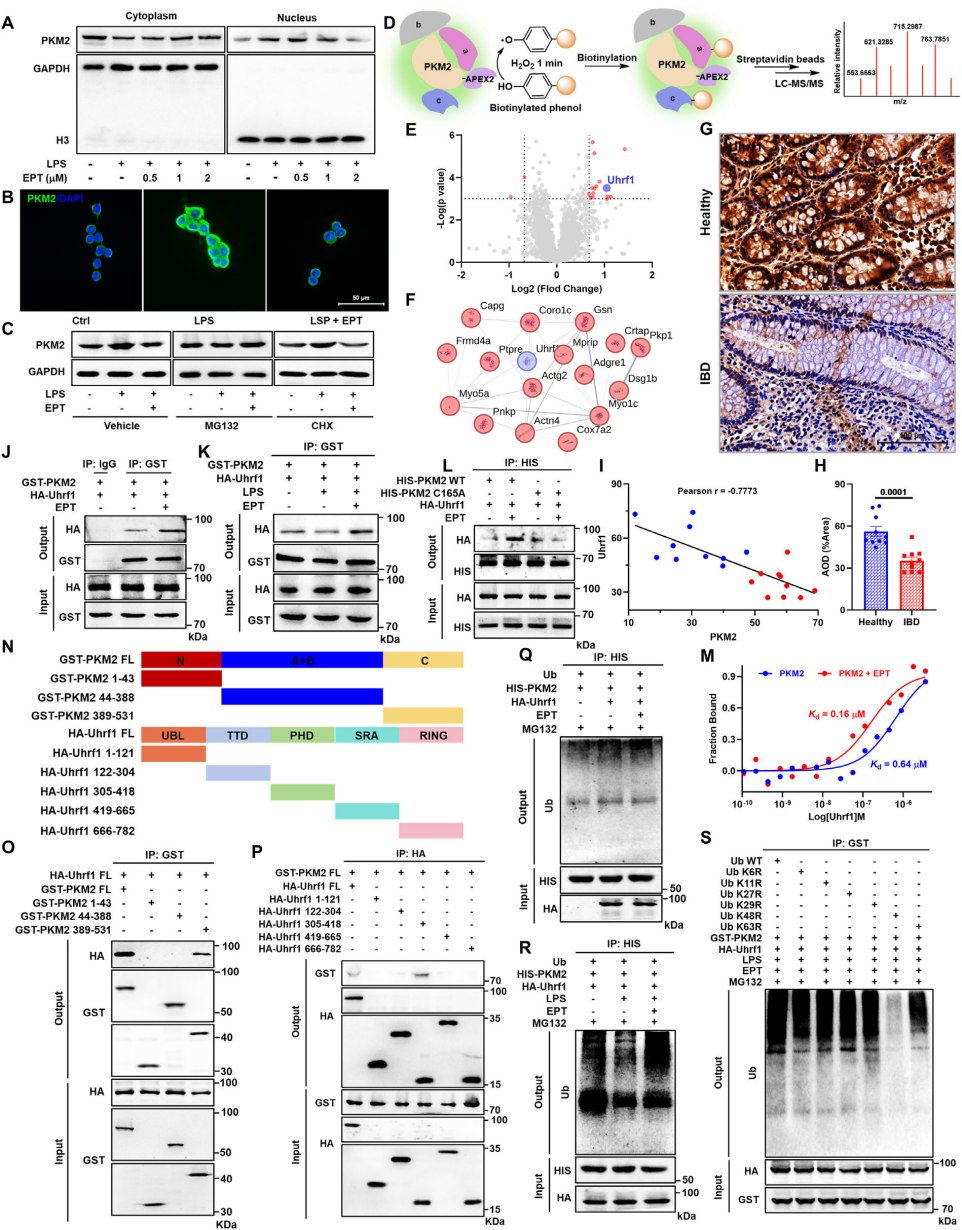
EPT promoted Uhrf1‐mediated PKM2 ubiquitination and degradation. (A) EPT blocked the nuclear translocation of PKM2; (B) PKM2 staining plot; (C) Effects of EPT toward PKM2 in the presence of CHX or MG132 in LPS‐mediated cells; (D) Workflow schematic for APEX2‐based spatial proteomics labeling system; (E) Uhrf1 was the key interaction protein of PKM2; (F) Protein‐protein interaction network; (G, H) Uhrf1 was downregulated in IBD patients; (I) Uhrf1 and PKM2 displayed the negative correlation; (J, K) EPT promoted the binding of PKM2 with Uhrf1 in the presence or absence of LPS; (L) PKM2 C165A mutation abolished effect of AB toward the binding of PKM2 with Uhrf1; (M) MST plot of PKM2 with Uhrf1 in the presence or absence of EPT; (N) Schematic shows three domains of PKM2 and five domains of Uhrf1; (O) Co‐IP result indicated that PKM2 interacted with PHD domain of Uhrf1; (P) Co‐IP result indicated that Uhrf1 interacted with C‐terminal domain of PKM2; (Q, R) Uhrf1 mediated the ubiquitination of PKM2 in the presence or absence of LPS; (S) Ubiquitination of PKM2 occurred through K48‐linked polyubiquitin chain formation.

To indicate their binding region, domain mapping of the Uhrf1‐PKM2 interaction was performed by constructing Uhrf1 truncates spanning its UBL, TTD, PHD, SRA, and RING domains alongside PKM2 conserved regions covering N, A+B, and C (Figure 7N). Co‐IP assays revealed that PKM2 directly interacted with Uhrf1's PHD domain (aa 305–418, Figure 7O), while Uhrf1 bound to the C‐terminal region (aa 389–531, Figure 7P) of PKM2, identifying these domains as critical for their mutual recognition. As visualized in Figure 7Q–S, Uhrf1‐mediated K48‐specific ubiquitination of PKM2 was observed, with EPT significantly potentiating this modification under both basal and LPS‐stimulated conditions. Targeted ubiquitin mutagenesis showed that the Lys48Arg (K48R) mutation abolished the ubiquitination of PKM2, revealing absolute dependence on K48 linkage (Figure 7S). These findings established that Uhrf1‐mediated K48‐linked polyubiquitination directed PKM2 toward proteasomal degradation.

### 2.9 EPT Depended on Uhrf1‐Mediated PKM2 Ubiquitination to Exert Its Protective Effect

To investigate the role of Uhrf1 in the anti‐inflammatory effect of EPT, Uhrf1‐specific siRNA plasmid for knockdown was transfected into cells, contributing to a decrease of mRNA and protein expression (Figure 8A). In complementary experiments, Uhrf1 restoration through exogenous overexpression significantly elevated both transcript and protein expression levels (Figure 8F). *Uhrf1* knockdown exacerbated LPS or LPS/NIG‐induced inflammatory response and pyroptosis, as evidenced by elevated mRNA levels of *iNOS*, *IL‐1β*, and *COX‐2* (Figure 8B), along with increased expressions of iNOS, COX‐2, pro IL‐1β, IL‐1β, and NLRP3 (Figure 8C,D). Furthermore, *Uhrf1* knockdown promoted the nuclear translocation of PKM2 in LPS‐mediated PKM2 overexpression cells (Figure 8E). Notably, Uhrf1 depletion abolished the protective efficacy of EPT in PKM2‐overexpressing cells (Figure 8B–E). In contrast, *Uhrf1* overexpression attenuated inflammation and pyroptosis triggered by LPS or LPS/NIG, which was supported by experiments of Western blot, PCR, and immunofluorescence staining (Figure 8G–J), and EPT displayed an extra anti‐inflammatory and anti‐pyroptotic effect in *PKM2* and *Uhrf1*‐overexpressing cells (Figure 8F–H). These findings collectively demonstrated that EPT's protective mechanism relied on Uhrf1‐dependent ubiquitination of PKM2.

**FIGURE 8:**
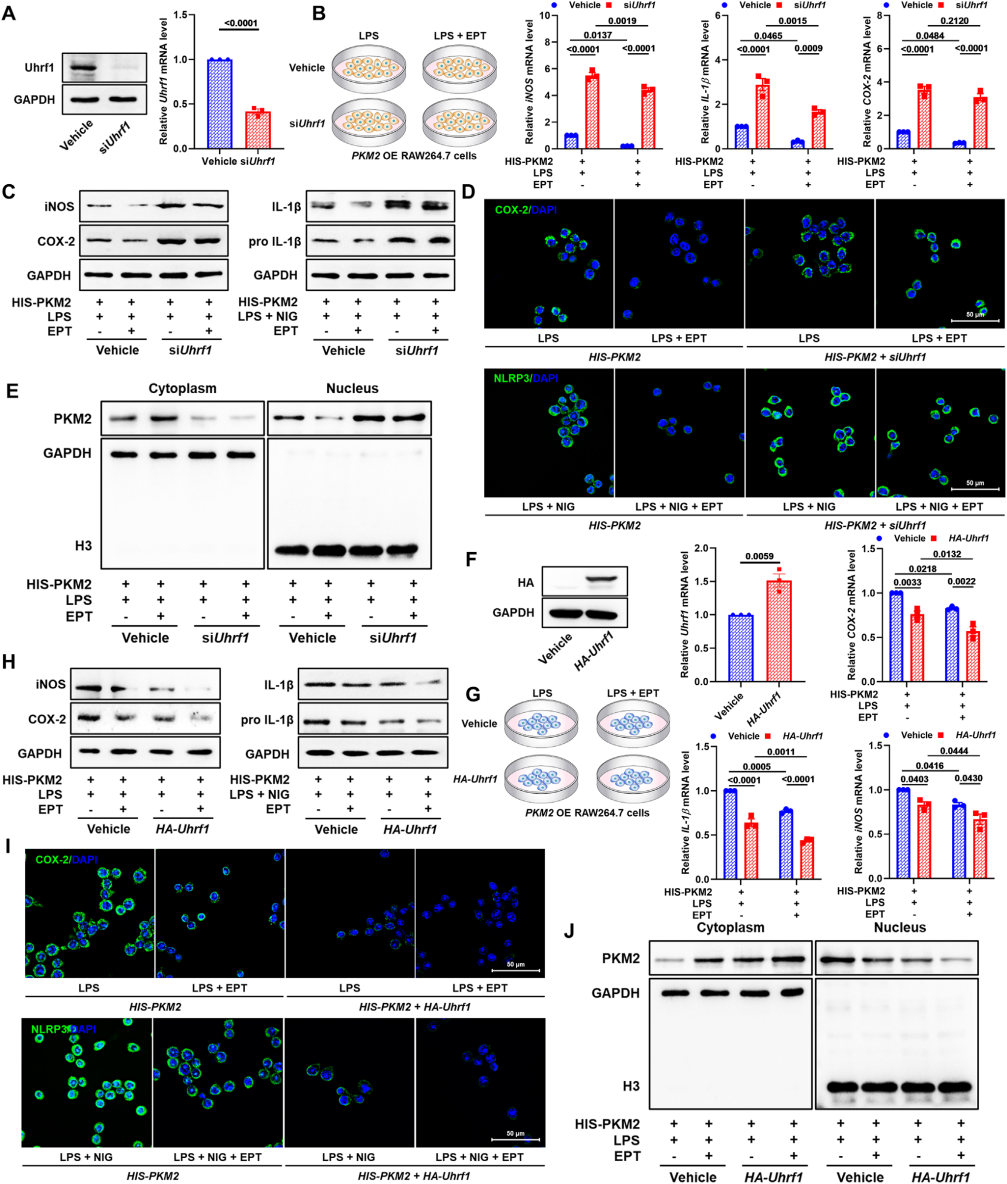
Anti‐inflammatory effects of EPT depended on Uhrf1‐mediated PKM2 ubiquitination. (A) Uhrf1 knockdown verification (*n* = 3); (B) *Uhrf1* knockdown weakened effects of EPT toward mRNA levels of *IL‐1β*, *iNOS*, and *COX‐2* in *PKM2* overexpression cells (*n* = 3); (C) *Uhrf1* knockdown weakened effects of EPT toward expressions of iNOS, COX‐2, IL‐1β, and pro IL‐1β in *PKM2* overexpression cells; (D) COX‐2 and NLRP3 staining plots; (E) *Uhrf1* knockdown led to the nuclear translocation of PKM2 in LPS‐mediated PKM2 overexpression cells; (F) *Uhrf1* overexpression verification (*n* = 3); (G) *Uhrf1* overexpression enhanced effects of EPT toward mRNA levels of *IL‐1β*, *iNOS*, and *COX‐2* in *PKM2* overexpression cells (*n* = 3); (H) *Uhrf1* overexpression enhanced effects of EPT toward expressions of iNOS, COX‐2, IL‐1β, and pro IL‐1β in *PKM2* overexpression cells; (I) COX‐2 and NLRP3 staining plots; (J) *Uhrf1* overexpression suppressed the nuclear translocation of PKM2 in LPS‐mediated PKM2 overexpression cells.

### 2.10 *PKM2* Genetic Knockdown Diminished the Anti‐IBD Effect of EPT In Vivo

To determine whether the protective effects of EPT toward IBD in vivo were dependent on PKM2, we injected lentiviruses with pLVX‐shPKM2 into mice for twice, based on the schematic diagram (Figure 9A), and successfully constructed *PKM2* genetic knockdown mice (Figure S21). Its knockdown ameliorated the reduction of body weight and the increase of rectal bleeding, diarrhea, and DAI scores in DSS‐mediated mice (Figure 9B and Figure S22). Meanwhile, the inflammatory cell infiltration and preserved intestinal crypt architecture were repressed (Figure 9C) in DSS‐induced IBD mice after *PKM2* knockdown, and its knockdown attenuated the shortening of DSS‐mediated colon length and gastrointestinal hemorrhage as well (Figure 9D,E). These improvements correlated with elevated occludin expression (Figure S23) and decreased mRNA levels of pro‐inflammatory cytokines *IL‐6*, *IL‐1α*, *IL‐1β*, *CCL5*, and *CXCL10* following DSS exposure (Figure 9F). Notably, EPT administration provided no additional therapeutic benefits in PKM2‐knockdown IBD mice (Figure 9B–F), confirming that EPT's anti‐IBD effects are dependent on PKM2.

**FIGURE 9:**
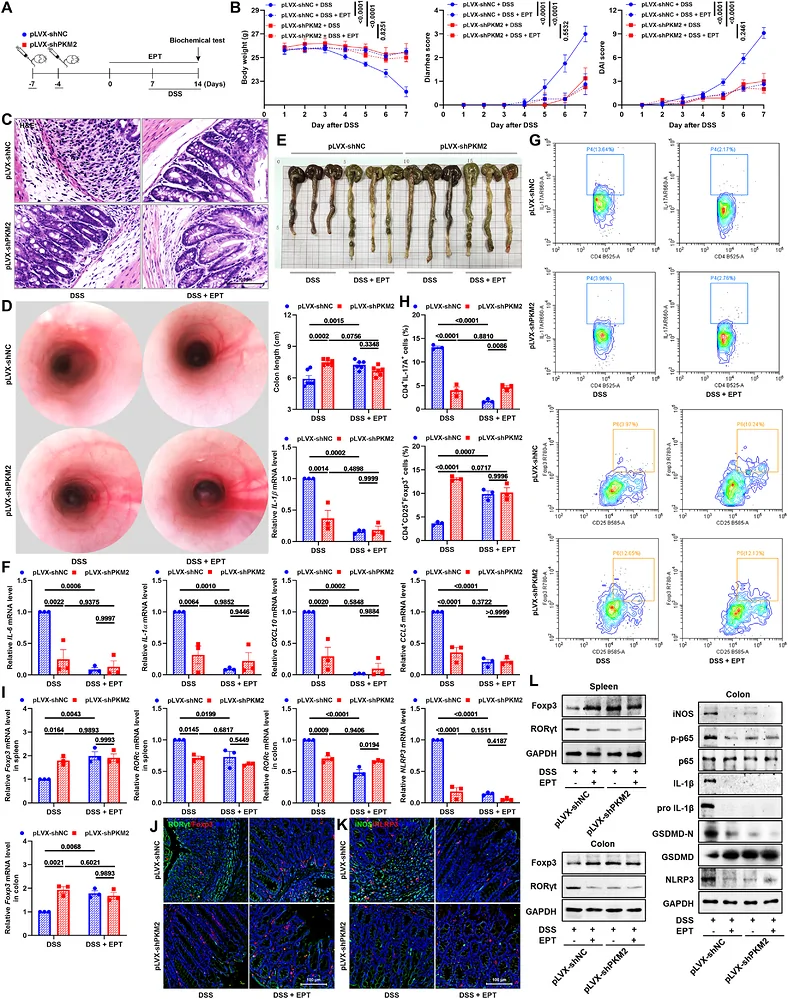
*PKM2* genetic knockdown abolished the anti‐IBD effect of EPT in vivo. (A) Schematic diagram for *PKM2* knockdown experiments; (B) Body weight, diarrhea and DAI score (*n* = 8); (C) H&E staining plot; (D) Colonoscopy plots; (E) Gross colon morphology and length (*n* = 6); (F and I) *PKM2* KO abolished effects of EPT toward mRNA levels of *IL‐1β*, *CCL5*, *IL‐6*, *IL‐1α*, *CXCL10*, *NLRP3, Foxp3*, and *RORc* (*n* = 3); (G, H) Flow cytometry results of Th17 (CD4^+^IL‐17A^+^) and Treg (CD4^+^CD25^+^Foxp3^+^) cells (*n* = 3); (J, K) RORγt/Foxp3 and iNOS/NLRP3 staining plots; (L) *PKM2* knockdown abolished effects of EPT toward expressions of RORγt, Foxp3, iNOS, p‐p65, p65, IL‐1β, pro IL‐1β, GSDMD‐N, GSDMD, and NLRP3.

### 2.11 *PKM2* Genetic Knockdown Eliminated EPT‐Mediated Restoration of Th17/Treg Balance and Suppression of Inflammatory Responses

Finally, we explored the protective effects of EPT on Th17/Treg immune balance and inflammatory response in wild‐type and *PKM2* knockdown mice. Genetic ablation of PKM2 significantly reduced splenic Th17 cell populations (CD4^+^IL‐17A^+^) while expanding Treg populations (CD4^+^CD25^+^Foxp3^+^), as confirmed by flow cytometry (Figure 9G,H, and Figure S24). These immunological shifts correlated with suppressed *RORc* expression and enhanced *Foxp3* levels at both mRNA and protein levels in splenic tissues (Figure 9I,J), with analogous findings observed in colonic tissues through immunofluorescence, Western blot, and qPCR analyses (Figure 9I,J,L). Notably, EPT did not have any extra effects for these indexes in DSS‐mediated *PKM2* knockdown mice (Figures 9G–J, L, and Figure S24). For inflammation, NF‐κB and NLRP3 pathways were also inactivated after *PKM2* knockdown in vivo (Figure 9K,L), and protective effects of EPT were diminished after its genetic knockdown in vivo. Collectively, these data established PKM2 as an essential mediator of EPT's therapeutic efficacy in IBD.

## 3 Discussion

IBD is a chronic inflammatory condition of the gastrointestinal tract characterized by progressive mucosal injury and ulcerative lesions, presenting as a significant public health concern worldwide [7]. Accumulating evidence demonstrated that immune imbalance, especially Th17 and Treg cells, resulted in inflammation, and they were associated with the course of IBD [33]. Th17 cells secrete pro‐inflammatory cytokines like IL‐17A and IL‐22, contributing to intestinal inflammation and tissue damage by activating NF‐κB and NLRP3 pathways [34], as evidenced in IBD patients where an increased presence of these cells in the intestinal mucosa correlates with elevated cytokine levels reflecting disease severity [35]. On the contrary, the proportion of Treg cells, responsible for anti‐inflammatory effects, is decreased in IBD patients [36]. Similarly, our scRNA‐seq analysis for the healthy and IBD patients indicated dysregulation of the NF‐κB/NLRP3 signaling and imbalance of Th17/Treg immune cells contributed to the inflammatory pathogenesis in IBD.

In recent years, advances in chemical analysis and molecular biology techniques have enabled the discovery of numerous potential drug candidates from natural products and TCM, injecting new vitality into pharmaceutical research [21]. Among them, the most representative case is the discovery of artemisinin from *Artemisia annua* [37], which has saved millions of lives and encouraged scientists to pursue innovative drug research through natural products and traditional Chinese medicines. Aucklandiae Radix, documented in the Chinese Pharmacopoeia for its ability to strengthen intestinal function and alleviate diarrhea [7], is widely utilized in clinical practice for managing various digestive disorders [32]. Recent research from our group demonstrated that Aucklandiae Radix exerted therapeutic effects against IBD by reducing the accumulation of intestinal macrophages and neutrophils [7]. This treatment further maintains intestinal immune homeostasis through modulation of the Treg/Th17 ratio [7]. In this study, by screening an in‐house Aucklandiae Radix library, we discovered the high‐efficacy lead EPT against IBD, revealing this medicinal plant as a rich reservoir for drug development.

Target fishing technologies have witnessed rapid advancements in recent years [26, 27], with innovative methods, such as protein microarray chips, affinity chromatography, thermal proteomics, solvent‐induced protein precipitation, and the biotin‐avidin system, being extensively utilized by chemical biologists [26, 27, 30]. These cutting‐edge approaches have facilitated the successful identification of molecular targets for various natural products, including kurarinone, wedelolactone, 1‐*O*‐acetyl‐4*R*,6*S*‐britannilactone, and 8*β*‐hydroxy‐*α*‐cyclocostunolide [28, 29, 30]. In this study, we synthesized a biotin‐labeled EPT to identify PKM2 as its cellular direct target with a *K*_d_ value of 106 nM using the biotin‐avidin technology. Using proteomic analysis, we demonstrated that EPT covalently bound to amino acid residue C165, which was supported by various chemical and biological methods. As one of four mammalian PK isoforms, PKM2 regulated critical biological pathways. Its overexpression was implicated in septic cardiomyopathy, cancer, renal fibrosis, and heart failure [38, 39, 40, 41]. PKM2 drove innate immunity by elevating pro‐inflammatory cytokines (e.g., IL‐1β and TNF‐α), while its tetramerization suppressed the STAT3/NLRP3 axis to protect against sepsis‐induced liver injury [14]. Consequently, PKM2 acts as a master regulator of inflammation, presenting a promising therapeutic target for inflammatory disorders. In our research, we identified that PKM2 was overexpressed in serums and colonic tissues of IBD patients. *PKM2* knockdown reduced inflammation and pyroptosis, while *PKM2* overexpression exacerbated these effects in vitro. Consistently, its knockdown significantly ameliorated disease severity in a DSS‐induced IBD murine model. Notably, EPT required *PKM2* to mediate its anti‐inflammatory effects, establishing PKM2 as a potential therapeutic target for IBD.

Despite evidence linking PKM2 to IBD, its mechanistic basis remains undefined. Leveraging APEX2 proximity labeling, a method enabling unbiased mapping of protein interactomes in combination with the biotin‐mediated pull‐down technique [26], we uncovered Uhrf1 as a critical binding partner of PKM2. Uhrf1, acting as an epigenetic coordinator, played a critical role in regulating key cellular processes, including replication, methylation, and repair of DNA [42]. Recently, a growing of evidence has demonstrated that Uhrf1 was associated with inflammation‐mediated diseases, such as hepatic fibrosis [43, 44]. For example, a study by Li et al., reported that Uhrf1 overexpression repressed macrophage M1 polarization and hepatic stellate cell activation to attenuate hepatic fibrosis [43], and its deficiency resulted in severe colitis by promoting hypomethylation of the TNF‐α promoter in a DSS‐treated mouse model [44]. Consistent with expectations, inflammation was reduced after *Uhrf1* rescue, whereas these effects were exacerbated after its knockdown in LPS‐mediated *PKM2* overexpression cells. Structural organization of Uhrf1 revealed five key functional domains, and Uhrf1‐mediated ubiquitination of various proteins since its RING domain possesses an intrinsic E3 ubiquitin ligase activity [45]. For example, Uhrf1 induces the ubiquitination of HP1β during S‐phase [46]. Furthermore, Qi and co‐workers revealed that Uhrf1 interacted with EG5 and promoted the ubiquitination of the latter to control mitotic spindle architecture and chromosome behavior [47]. In this study, we demonstrated that Uhrf1 bound to the C‐terminal domain of PKM2 via its PHD domain, triggering K48‐linked ubiquitination and subsequent proteasomal degradation of PKM2 (Figure 10). Notably, pharmacological inhibition of PKM2 using EPT enhanced its interaction with Uhrf1, leading to further suppression of PKM2 expression and blockade of its nuclear translocation for treating IBD.

**FIGURE 10:**
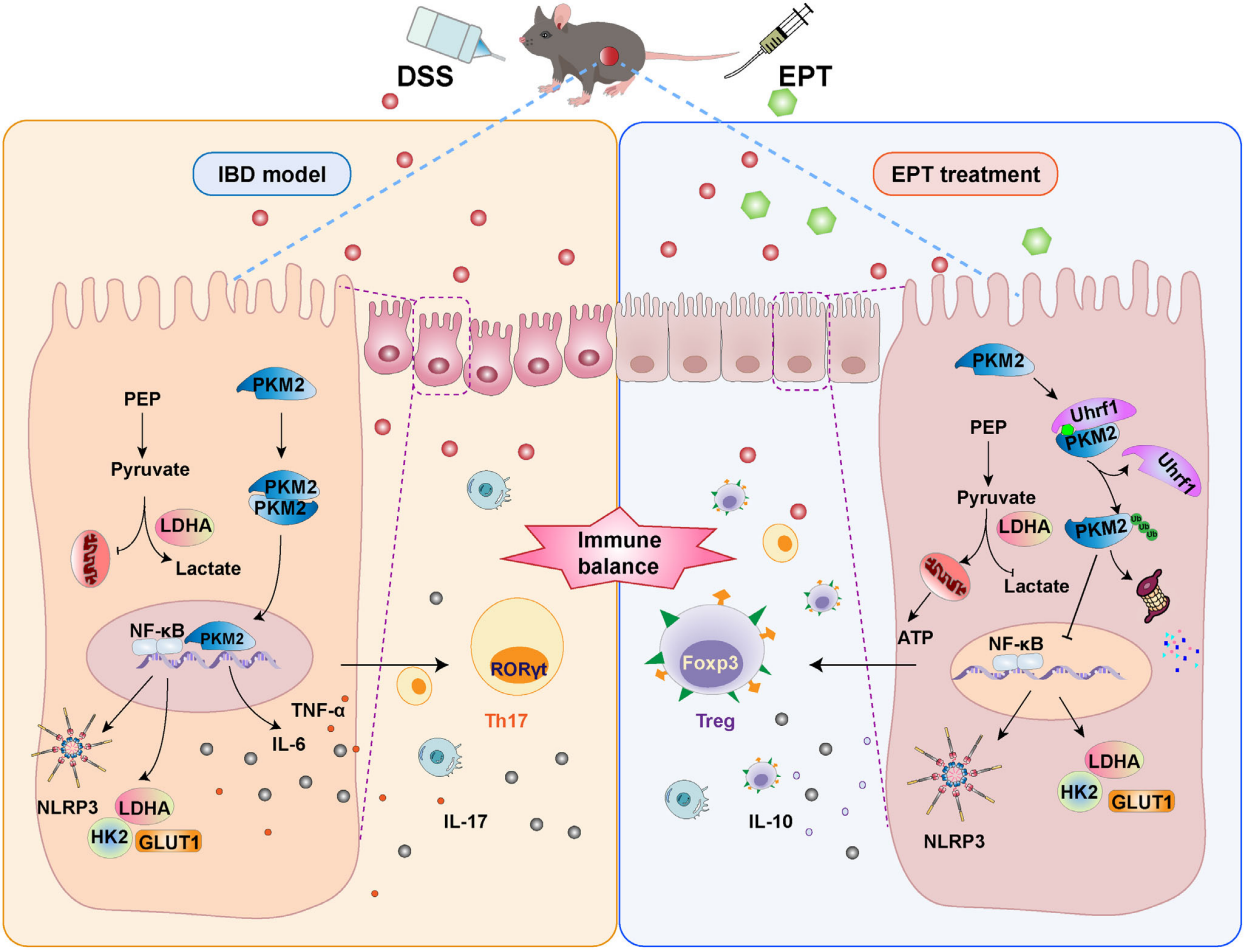
Mechanism of Uhrf1‐mediated PKM2 ubiquitination to alleviate IBD.

## 4 Conclusion

In summary, our study provided the first evidence that Uhrf1 orchestrated K48‐linked ubiquitination of PKM2, targeting it for proteasomal degradation—a process critical for mitigating inflammation and pyroptosis in IBD. The small‐molecule EPT demonstrated high‐affinity binding (*K*_d_ = 106 nM) via covalent modification at PKM2's C165 residue, which paradoxically enhanced Uhrf1‐PKM2 complex formation while preventing nuclear accumulation of PKM2. Notably, these findings positioned EPT as a versatile chemical probe for dissecting PKM2's multifaceted roles across physiological systems and disease states.

## 5 Experimental Section

### 5.1 Materials

The primary antibodies occludin (27260‐1‐AP), RORγt (29910‐1‐AP), Foxp3 (22228‐1‐AP), GSDMD (66387‐1‐Ig), LDHA (19987‐1‐AP), GLUT1 (21829‐1‐AP), Mfn1 (13798‐1‐AP), Mfn2 (12186‐1‐AP), Opa1 (27733‐1‐AP), Fis1 (10956‐1‐AP), TOM20 (A19403), COX‐2 (66351‐1‐Ig), iNOS (22226‐1‐AP), p‐p65 (AP0124), p65 (10745‐1‐AP), caspase 1 (22915‐1‐AP), IL‐1β (26048‐1‐AP), ASC (AB309497), NLRP3 (68102‐1‐Ig), PKM2 (60268‐1‐Ig), Drp1 (12957‐1‐AP), H3 (17168‐1‐AP), HA (51054‐1‐AP), GST (80006‐1‐AP), HIS (66005‐1‐AP), Ub (10201‐1‐AP), Uhrf1 (21402‐1‐AP), and GAPDH (60004‐1‐Ig) were purchased from Abcam (Shanghai, China), Abclonal (Wuhan, China) and Proteintech (Wuhan, China), respectively.

### 5.2 Lentiviral Vector Preparation and Titer Determination

Lentivirus was prepared in HEK293T cells by PEI‐mediated transfection. The transfer plasmid, psPAX2 packaging plasmid, and pMD2.G envelope plasmid were co‐transfected at a mass ratio of 4:3:1, with a total DNA dosage of 20 µg and a PEI‐to‐DNA ratio of 2.5:1. The viral supernatant was collected at 48 h post‐transfection, filtered through a 0.45 µm membrane, aliquoted, and stored at −80°C. Subsequently, HEK293T cells were infected with gradient volumes of lentiviral supernatant. After infection, cellular genomic DNA was extracted, and the copy numbers of viral HIV sequences and the internal reference gene GAPDH were detected via qPCR. The lentiviral titer was calculated using dual standard curves and the corresponding formula.

### 5.3 Animals

All studies were conducted in accordance with protocols approved by the Animal Care and Use Committee of Tianjin University of Traditional Chinese Medicine (Approval No. TCM‐LAEC2023208s1550) using male C57BL/6J mice (20‐25 g). IBD was induced by administering 3% DSS in the drinking water. EPT was administered by oral gavage at doses of 5, 10, and 20 mg/kg (dissolved in 10% β‐cyclodextrin), with an equivalent volume of the vehicle solution serving as the control. In the gene knockdown experiment, lentiviral vectors (1 × 10^8^ TU/mL) were injected via the tail vein for two times, based on the schematic diagram (Figure 9A), before EPT treatment. EPT treatment (20 mg/kg, i.g.) was initiated for 14 days, followed by induction of colitis with 3% DSS in drinking water starting at eighth day for 7 days. Colon and spleen tissue were collected for further experiments.

### 5.4 H&E, Immunohistochemistry, and Immunofluorescence Analysis

Colon tissues were fixed in 4% PFA, embedded in paraffin, and sectioned for analysis. Deparaffinized and rehydrated sections were used for H&E staining. For immunohistochemistry (IHC), antigen‐retrieved sections were blocked and incubated with primary antibodies at 4°C overnight, followed by secondary antibodies, DAB, and hematoxylin counterstaining. For immunofluorescence (IF), tissue sections following antigen retrieval were sequentially incubated with primary antibodies and fluorophore‐conjugated secondary antibodies, and subsequently stained with DAPI. All sections were imaged under a Leica DM6B thunder microscope.

### 5.5 Flow Cytometry

Single‐cell suspensions were prepared from mouse spleens by mechanical dissociation in ice‐cold PBS, followed by erythrocyte lysis, filtration, and counting. Primary spleen cells were cultured in 1640 medium supplemented with 10% fetal bovine serum and 1% antibiotic‐antimycotic solution. For immunophenotyping, cells were stained for Treg (CD4, CD25, and Foxp3) or Th17 markers (CD4 and IL‐17A). In the flow cytometry experiment, after gating on CD4^+^ T cells using CD4 antibody, Treg cells were subsequently identified through CD25 and Foxp3 antibodies, while Th17 cells were characterized by IL‐17A antibody. RAW264.7 cells, pretreated with EPT (2 µM) for 1 h and stimulated with LPS (500 ng/mL) for 24 h, were processed with a protein transport inhibitor added 6 h before harvesting. All samples were fixed, blocked, and incubated with fluorochrome‐conjugated primary antibodies or primary antibodies followed by FITC‐conjugated secondary antibodies. Data were acquired on a CytoFlex flow cytometer (Beckman, USA).

### 5.6 Cell Culture and Treatment

RAW264.7 and HEK293T cells were maintained in their respective complete culture media at 37°C in a humidified incubator with 5% CO_2_. According to the experimental design, cells were pretreated with EPT (0.5, 1, and 2 µM) for 1 h, followed by stimulation with LPS (500 ng/mL). For experiments requiring overexpression or siRNA transfection, cells were serum‐starved for 2 h prior to transfection, and transfections were performed using PEI or Lipofectamine 2000. The medium was replaced with complete medium 6 h post‐transfection. For ubiquitination assays, the proteasome inhibitor MG132 was added at a final concentration of 10 µM to prevent proteasome‐mediated protein degradation.

### 5.7 Mitochondrial Membrane Potential Assay

JC‐1 dye and Hoechst33342 were prepared as working solutions in JC‐1 assay buffer. After incubation with cells at 37°C for 30 min, the dye solution was removed. Coverslips were mounted with anti‐fade mounting medium and imaged using a Leica DM6B thunder system.

### 5.8 Quantitative Real‐Time PCR

Total RNA was extracted using the Trizol method, and its concentration and purity were assessed using a NanoPhotometer N50 spectrophotometer (Implen, Germany). RNA was reverse transcribed into cDNA, and quantitative PCR was performed using an Applied Biosystems QuantStudio 1 system (Applied Biosystems, USA) with *GAPDH* as the internal reference gene.

### 5.9 Western Blot

Total protein was isolated with a lysis buffer (1% PMSF), and protein concentration was determined by the BCA method. Equal amounts of protein were separated by 7.5%–15% SDS‐PAGE and then transferred to a polyvinylidene fluoride (PVDF) membrane. The membrane was incubated with primary antibodies at 4°C, and subsequently with secondary antibodies at room temperature. Target proteins were detected employing an enhanced chemiluminescence (ECL) system, with band intensity quantified using ImageJ software.

### 5.10 OXPHOS and Glycolysis Assay

Seahorse experiments were performed using an Agilent Seahorse XF96 analyzer according to protocols of the OCR and PER corresponding kits. Following 12 h treatments with LPS and EPT as previously reported, cells underwent three PBS washes and were equilibrated in Seahorse XF base medium. For PER measurements, rotenone/antimycin A and 2‐DG were administered at concentrations of 0.5 µM and 50 mM, respectively. In the OCR assay, oligomycin, FCCP, and rotenone/antimycin A were sequentially injected at final concentrations of 2, 2, and 0.5 µM. All data were processed using Wave software for analysis.

### 5.11 Cellular Thermal Shift Assay (CETSA)

Cell lysates were incubated with EPT (50 µM) or DMSO at room temperature for 1 h, then aliquoted into five equal parts and heated at specified temperatures (46°C–62°C). Subsequently, detect the expression levels of target proteins through Western blot analysis.

### 5.12 Drug Affinity Responsive Target Stability (DARTS)

Cell lysates were incubated with EPT at varying concentrations (25, 50, and 100 µM) or DMSO at room temperature for 2 h, followed by digestion with pronase (5 µg/mL) at 37°C. Subsequently, assess the expression levels of target proteins using Western blot.

### 5.13 Pull‐Down Assay

Cell lysates were preincubated with IAA or EPT at 4°C for 3 h, followed by overnight incubation with Bio‐EPT. Proteins were enriched using streptavidin magnetic beads and subsequently separated by SDS–PAGE. The enriched proteins were analyzed by immunoblotting or silver staining, and protein identification was performed by LC‐MS/MS.

### 5.14 Immunoprecipitation (IP)

Equal amounts of cell lysates were incubated with IgG, GST, or HA‐specific antibodies at 4°C for 1 h, followed by overnight incubation with Protein A/G magnetic beads. After extensive washing to remove nonspecific binding, the enriched proteins were eluted by boiling in 1×loading buffer and subsequently analyzed by immunoblotting.

### 5.15 APEX2 Proximity Labeling Assay

RAW264.7 cells were transfected with an APEX2‐PKM2 plasmid using Lipofectamine 2000. Following EPT and LPS treatment, proximity‐dependent biotinylation was conducted by incubating cells with biotin‐phenol, followed by a brief H_2_O_2_ stimulation. Biotinylated proteins were harvested, affinity‐purified using streptavidin magnetic beads, and subsequently identified by LC‐MS/MS.

### 5.16 Microscale Thermophoresis (MST) Assay

PKM2 was labeled with a fluorescent dye using the NHS‐labeling Kit. The labeled protein was incubated with a series of EPT concentrations in standard buffer, and the dissociation constant (*K*_d_) was measured using the Monolith instrument (NanoTemper Technologies, Munich, Germany).

### 5.17 scRNA‐seq

scRNA‐seq data of colonic tissues from human donors were obtained from the publicly available database GSE282122. Data analyses were performed in the R environment using the Seurat package (v4.5.1). Following standard workflows, raw data were subjected to quality control to remove cells expressing fewer than 200 or more than 6000 genes, cells with mitochondrial gene proportions exceeding 10%, and genes detected in fewer than three cells. To further minimize technical noise, ribosomal and erythrocyte‐related genes were excluded, and only cells with total UMI counts between 500 and 60 000 were retained. The filtered data were normalized and scaled, and dimensionality reduction and clustering were conducted using PCA and uniform manifold approximation and projection (UMAP). Batch effects among samples were corrected using the Harmony algorithm. After cell‐type annotation, differential expression analysis (DEG) was performed to identify key genes associated with UC progression, and pathways, together with metabolic enrichment analyses, were conducted to reveal potential biological functional differences.

### 5.18 Statistical Analysis

Statistical significance was assessed with GraphPad Prism 10 software (GraphPad Software Inc., USA). Depending on the experimental design, comparisons were made using the Student's *t*‐test, one‐way ANOVA, or two‐way ANOVA. Where applicable, Tukey's post hoc test was conducted thereafter. Differences were deemed significant at *p* < 0.05, and data are presented as mean ± SEM.

## Author Contributions

**Juan Zhang**: Writing – original draft, funding acquisition, Writing – review and editing, investigation, formal analysis. **Feng Qiu**: project administration. **Xin‐Rong Xu**: investigation, writing – original draft. **Jing‐Hui Du**: investigation. **Cheng‐Peng Sun**: investigation, funding acquisition, writing – original draft, writing – review and editing. **Qi‐Meng Zhu**: investigation, visualization. **Christophe Morisseau**: investigation, visualization. **Xi‐Peng Shang**: investigation, supervision. **Hui‐Lin Zhang**: investigation. **Xin‐Yuan Li**: investigation, visualization.

## Funding

This work was supported by National Natural Science Foundation of China (No. 82574688), Support Program for Young Faculty's Research and Innovation Capability Development in Tianjin Higher Education Institutions Initiated by the Ministry of Education, Outstanding Youth Foundation of Tianjin (No. 25JCJQJC00180), Natural Science Foundation of Tianjin (No. 25JCYBJC00620), and Young Scientific and Technological Talents (Level Two) in Tianjin (No. QN20230212).

## Conflicts of Interest

The authors declare no conflicts of interest.

## Supporting information




**Supporting File 1**: advs77403‐sup‐0001‐SuppMat.docx.

## Data Availability

All data generated or analyzed during this study are included in this article and its supplementary files.

## References

[1] T. Kobayashi and T. Hibi , “Improving IBD Outcomes in the Era of Many Treatment Options,” Nature Reviews Gastroenterology & Hepatology 20 (2023): 79–80, 10.1038/s41575-022-00738-z. PMC9838431 36635556

[2] J. Lu , X. Shen , H. Li , and J. Du , “Recent Advances in Bacteria‐Based Platforms for Inflammatory Bowel Diseases Treatment,” Exploration 4 (2024): 20230142, 10.1002/EXP.20230142. PMC11491310 39439496

[3] J. Yang , S. Tan , S. Ge , et al., “Cyanobacteria–Probiotics Symbionts for Modulation of Intestinal Inflammation and Microbiome Dysregulation in Colitis,” Proceedings of the National Academy of Sciences 121 (2024): 2403417121, 10.1073/pnas.2403417121. PMC11670216 39680761

[4] S. C. Ng , H. Y. Shi , N. Hamidi , et al., “Worldwide Incidence and Prevalence of Inflammatory Bowel Disease in the 21st Century: A Systematic Review of Population‐Based Studies,” The Lancet 390 (2017): 2769–2778, 10.1016/S0140-6736(17)32448-0. 29050646

[5] S. Coward , F. Clement , E. I. Benchimol , et al., “Past and Future Burden of Inflammatory Bowel Diseases Based on Modeling of Population‐Based Data,” Gastroenterology 156 (2019): 1345–1353.e4, 10.1053/j.gastro.2019.01.002. 30639677

[6] G. R. Jones , M. Lyons , N. Plevris , et al., “IBD Prevalence in Lothian, Scotland, Derived by Capture–Recapture Methodology,” Gut 68 (2019): 1953–1960, 10.1136/gutjnl-2019-318936. PMC6839733 31300515

[7] Y. L. Feng , X. R. Xu , Q. M. Zhu , et al., “Aucklandiae Radix Targeted PKM2 to Alleviate Ulcerative Colitis: Insights From the Photocrosslinking Target Fishing Technique,” Phytomedicine 134 (2024): 155973, 10.1016/j.phymed.2024.155973. 39241384

[8] X. Yang , G. Li , P. Lou , et al., “Excessive Nucleic acid R‐Loops Induce Mitochondria‐Dependent Epithelial Cell Necroptosis and Drive Spontaneous Intestinal Inflammation,” Proceedings of the National Academy of Sciences 121 (2024): 2307395120, 10.1073/pnas.2307395120. PMC10769860 38157451

[9] D. Hou , T. Yu , X. Lu , et al., “Sirt2 inhibition Improves Gut Epithelial Barrier Integrity and Protects Mice From Colitis,” Proceedings of the National Academy of Sciences 121 (2024): 2319833121, 10.1073/pnas.2319833121. PMC11066986 38648480

[10] P. Wangchuk , K. Yeshi , and A. Loukas , “Ulcerative Colitis: Clinical Biomarkers, Therapeutic Targets, and Emerging Treatments,” Trends in Pharmacological Sciences 45 (2024): 892–903, 10.1016/j.tips.2024.08.003. 39261229

[11] M. Coskun , S. Vermeire , and O. H. Nielsen , “Novel Targeted Therapies for Inflammatory Bowel Disease,” Trends in Pharmacological Sciences 38 (2017): 127–142, 10.1016/j.tips.2016.10.014. 27916280

[12] C. Liu , C. Liu , and R. Fu , “Research Progress on the Role of PKM2 in the Immune Response,” Frontiers in Immunology 13 (2022): 936967, 10.3389/fimmu.2022.936967. PMC9365960 35967360

[13] D. Pavlenko , J. Tamargo‐Azpilicueta , H. Nudelman , et al., “Isoform‐Specific Regulation of PKM by Acetylation,” Proceedings of the National Academy of Sciences 122 (2025): 2527086122, 10.1073/pnas.2527086122. PMC12685146 41289402

[14] B. Wu , X. Yu , Y. Li , et al., “Forsythoside E Alleviateds Liver Injury by Targeting PKM2 Tetramerization to Promote Macrophage M2 Polarization,” Advanced Science 13 (2025): e14514, 10.1002/advs.202514514. PMC12866822 41236855

[15] Z. Zhang , X. Deng , Y. Liu , Y. Liu , L. Sun , and F. Chen , “PKM2, Function and Expression and Regulation,” Cell & Bioscience 9 (2019): 52, 10.1186/s13578-019-0317-8. PMC6595688 31391918

[16] Z. Liu , Y. Le , H. Chen , J. Zhu , and D. Lu , “Role of PKM2‐Mediated Immunometabolic Reprogramming on Development of Cytokine Storm,” Frontiers in Immunology 12 (2021): 748573, 10.3389/fimmu.2021.748573. PMC8572858 34759927

[17] W. Yang , Y. Zheng , Y. Xia , et al., “ERK1/2‐Dependent Phosphorylation and Nuclear Translocation of PKM2 Promotes the Warburg Effect,” Nature Cell Biology 14 (2012): 1295–1304, 10.1038/ncb2629. PMC3511602 23178880

[18] L. E. A. Damasceno , D. S. Prado , F. P. Veras , et al., “PKM2 Promotes Th17 Cell Differentiation and Autoimmune Inflammation by Fine‐Tuning STAT3 Activation,” Journal of Experimental Medicine 217 (2020): 20190613. 10.1084/jem.20190613 PMC7537396 32697823

[19] J. Wang , P. Yang , T. Yu , et al., “Lactylation of PKM2 Suppresses Inflammatory Metabolic Adaptation in Pro‐inflammatory Macrophages,” International Journal of Biological Sciences 18 (2022): 6210–6225, 10.7150/ijbs.75434. PMC9682528 36439872

[20] A. A. Almousa , M. Morris , S. Fowler , J. Jones , and J. Alcorn , “Elevation of Serum Pyruvate Kinase M2 (PKM2) in IBD and its Relationship to IBD Indices,” Clinical Biochemistry 53 (2018): 19–24, 10.1016/j.clinbiochem.2017.12.007. 29273328

[21] J. L. Huang , L. M. Wu , S. Q. Wu , et al., “A Small Molecule Targets LIC1 to Suppress Lung Tumor Growth by Inducing Autophagy,” Nature Chemical Biology 22 (2025): 459–470, 10.1038/s41589-025-02040-w. 41102409

[22] L. Xu , Z. Shao , X. Fang , et al., “Exploring Precision Treatments in Immune‐Mediated Inflammatory Diseases: Harnessing the Infinite Potential of Nucleic Acid Delivery,” Exploration 5 (2025): 20230165. 10.1002/EXP.20230165 PMC11875455 40040830

[23] J. Zhang , J. Yan , H. Dong , et al., “Dimeric Sesquiterpenoids with Anti‐Inflammatory Activities from Inula Britannica,” Chinese Journal of Natural Medicines 23 (2025): 961–971. 10.1016/S1875-5364(25)60931-9 40754377

[24] L. Yang , H. H. Xu , Q. Hong , et al., “Crocus Sativus L. Produces Anti‐Inflammatory Effects and Regulates the NLRP3–NF‐κB Pathway,” Acupuncture and Herbal Medicine 4 (2024): 375–385, 10.1016/j.apsb.2022.01.002.

[25] Z. Luo , F. Yin , X. Wang , and L. Kong , “Progress in Approved Drugs from Natural Product Resources,” Chinese Journal of Natural Medicines 22 (2024): 195–211. 10.1016/S1875-5364(24)60582-0 38553188

[26] J. Zhang , H. L. Zhang , X. R. Xu , et al., “Targeting PBK With Small‐Molecule 1‐ O ‐acetyl‐4 R ,6 S ‐Britannilactone for the Treatment of Neuroinflammation,” Proceedings of the National Academy of Sciences 122 (2025): 2502593122, 10.1073/pnas.2502593122. PMC12304890 40658838

[27] C. P. Sun , J. J. Zhou , Z. L. Yu , et al., “Kurarinone Alleviated Parkinson's Disease via Stabilization of Epoxyeicosatrienoic Acids in Animal Model,” Proceedings of the National Academy of Sciences 119 (2022): 2118818119. 10.1073/pnas.2118818119 PMC8892522 35217618

[28] J. Zhang , M. Zhang , X. K. Huo , et al., “Macrophage Inactivation by Small Molecule Wedelolactone via Targeting sEH for the Treatment of LPS‐Induced Acute Lung Injury,” ACS Central Science 9 (2023): 440–456, 10.1021/acscentsci.2c01424. PMC10037491 36968547

[29] J. Zhang , M. Zhang , Q. M. Zhu , et al., “Allosteric Regulation of Keap1 by 8β‐hydroxy‐α‐cyclocostunolide for the Treatment of Acute Lung Injury,” Acta Pharmaceutica Sinica B 14 (2024): 4174–4178, 10.1016/j.apsb.2024.06.025. PMC11413700 39309504

[30] J. Zhang , Z. L. Luan , X. K. Huo , et al., “Direct Targeting of sEH With Alisol B Alleviated the Apoptosis, Inflammation, and Oxidative Stress in Cisplatin‐Induced Acute Kidney Injury,” International Journal of Biological Sciences 19 (2023): 294–310, 10.7150/ijbs.78097. PMC9760444 36594097

[31] J. Zhang , W. H. Zhang , C. Morisseau , et al., “Genetic Deletion or Pharmacological Inhibition of Soluble Epoxide Hydrolase Attenuated Particulate Matter 2.5 Exposure Mediated Lung Injury,” Journal of Hazardous Materials 458 (2023): 131890, 10.1016/j.jhazmat.2023.131890. PMC10699546 37406527

[32] Z. Huang , C. Wei , K. Yang , Z. Yu , Z. Wang , and H. Hu , “Aucklandiae Radix and Vladimiriae Radix: A Systematic Review in Ethnopharmacology, Phytochemistry and Pharmacology,” Journal of Ethnopharmacology 280 (2021): 114372, 10.1016/j.jep.2021.114372. 34186101

[33] C. Danne , J. Skerniskyte , B. Marteyn , and H. Sokol , “Neutrophils: From IBD to the Gut Microbiota,” Nature Reviews Gastroenterology & Hepatology 21 (2024): 184–197, 10.1038/s41575-023-00871-3. 38110547

[34] Y. L. Wang , H. Zhu , Y. T. Pan , et al., “Dendritic Cell Mineralocorticoid Receptor Controls Blood Pressure by Regulating T Helper 17 Differentiation: Role of the Plcβ1/4–Stat5–NF‐κB Pathway,” European Heart Journal 46 (2025): 1335–1351, 10.1093/eurheartj/ehae670. 39498862

[35] I. Leonardi , I. H. Gao , W. Y. Lin , et al., “Mucosal Fungi Promote Gut Barrier Function and Social Behavior via Type 17 Immunity,” Cell 185 (2022): 831–846.e14, 10.1016/j.cell.2022.01.017. PMC8897247 35176228

[36] V. Mitsialis , S. Wall , P. Liu , et al., “Single‐Cell Analyses of Colon and Blood Reveal Distinct Immune Cell Signatures of Ulcerative Colitis and Crohn's Disease,” Gastroenterology 159 (2020): 591–608.e10. 10.1053/j.gastro.2020.04.074 PMC8166295 32428507

[37] S. L. Croft and S. Ward , “The Nobel Prize in Medicine 2015: Two Drugs that Changed Global Health,” Science Translational Medicine 7 (2015): 316ed14, 10.1126/scitranslmed.aad5868. 26631629

[38] M. A. Lorenzana‐Carrillo , K. Gopal , N. J. Byrne , et al., “TRIM35‐Mediated Degradation of Nuclear PKM2 Destabilizes GATA4/6 and Induces P53 in Cardiomyocytes to Promote Heart Failure,” Science Translational Medicine 14 (2022): abm3565, 10.1126/scitranslmed.abm3565. 36322626

[39] L. Ni , B. Lin , M. Shen , et al., “PKM2 Deficiency Exacerbates Gram‐Negative Sepsis‐Induced Cardiomyopathy via Disrupting Cardiac Calcium Homeostasis,” Cell Death Discovery 8 (2022): 496, 10.1038/s41420-022-01287-9. PMC9789059 36564378

[40] M. Wu , G. Jia , Y. Liu , et al., “PKM2 Controls Cochlear Development Through Lactate‐Dependent Transcriptional Regulation,” Proceedings of the National Academy of Sciences 122 (2025): 2410829122, 10.1073/pnas.2410829122. PMC11745320 39773029

[41] T. Xiang , X. Wang , S. Huang , et al., “Inhibition of PKM2 by Shikonin Impedes TGF‐β1 Expression by Repressing Histone Lactylation to Alleviate Renal Fibrosis,” Phytomedicine 136 (2025): 156324, 10.1016/j.phymed.2024.156324. 39700636

[42] M. Mancini , E. Magnani , F. Macchi , and I. M. Bonapace , “The Multi‐Functionality of UHRF1: Epigenome Maintenance and Preservation of Genome Integrity,” Nucleic Acids Research 49 (2021): 6053–6068, 10.1093/nar/gkab293. PMC8216287 33939809

[43] R. Li , Q. Tao , J. Zha , et al., “UHRF1 Ameliorates Liver Cirrhosis Through Suppressing Macrophage M1 Polarization by Catalyzing HMGA2 Promoter Methylation,” Biochemical Pharmacology 242 (2025): 117357, 10.1016/j.bcp.2025.117357. 40976429

[44] S. Qi , Y. Li , Z. Dai , et al., “Uhrf1‐Mediated Tnf‐α Gene Methylation Controls Proinflammatory Macrophages in Experimental Colitis Resembling Inflammatory Bowel Disease,” The Journal of Immunology 203 (2019): 3045–3053, 10.4049/jimmunol.1900467. 31611260

[45] L. Gu , Y. Fu , and X. Li , “Roles of Post‐Translational Modifications of UHRF1 in Cancer,” Epigenetics & Chromatin 17 (2024): 15. 10.1186/s13072-024-00540-y PMC11080273 38725075

[46] W. Qin , C. Steinek , K. Kolobynina , et al., “Probing Protein Ubiquitination in Live Cells,” Nucleic Acids Research 50 (2022): 125, 10.1093/nar/gkac805. PMC9757074 36189882

[47] X. Qi , Y. Liu , Y. Peng , et al., “UHRF1 Promotes Spindle Assembly and Chromosome Congression by Catalyzing EG5 Polyubiquitination,” Journal of Cell Biology 222 (2023): 202210093. 10.1083/jcb.202210093 PMC10510743 37728657

