# Supplementary material for: Uhrf1‐Mediated PKM2 Degradation via Ubiquitination Alleviates Inflammation and Pyroptosis in Inflammatory Bowel Disease
Source: Adv Sci (Weinh). 2026 Aug 25;13(61):e77403. doi: 10.1002/advs.77403 (PMC13505462; doi:10.1002/advs.77403)
Supplement: Supplementary file 1 [file ADVS-13-e77403-s001.docx]

Supplementary Materials for

**Uhrf1-mediated PKM2 degradation via ubiquitination alleviates inflammation and** **pyroptosis in inflammatory bowel disease**

Juan Zhang^1,+^, Xin-Rong Xu^1,+^, Hui-Lin Zhang^1,+^, Qi-Meng Zhu^1^, Xin-Yuan Li^1^, Xi-Peng Shang^2^, Jing-Hui Du^2^, Christophe Morisseau^3^, Feng Qiu^1^, Cheng-Peng Sun^1,^*

**Figure S1.** Rectal bleeding score in DSS-mediated IBD after EPT administration (mean ± SEM, n = 8);

**Figure S2** Effect of EPT toward Occludin expression (mean ± SEM, n = 3).

**Figure S3.** Effects of EPT toward mRNA levels of *IL-1α*, *CCL5*, *CXCL10*, and *NLRP3* (n = 3).

**Figure S4.** (A) Flow cytometry results of Th17 (CD4^+^IL-17A^+^) and Treg (CD4^+^CD25^+^Foxp3^+^) cells after defining the gate of CD4^+^ T cells by FITC-CD4 anti-body; (B) Effects of EPT toward expressions for RORγt and Foxp3 in colon (n = 3).

**Figure S5.** Effects of EPT toward *Foxp3*, *RORc*, and *IL-17A* mRNA level in spleen (n = 3).

**Figure S6.** (A) Quantitative data of RORγt and Foxp3 in spleen (n = 3); (B) Quantitative data of RORγt and Foxp3 in colon (n = 3).

**Figure S7.** Quantitative data of iNOS, p-p65/p65, IL-1β, pro IL-1β, cleaved caspase 1/caspase 1, ASC, and NLRP3 in DSS-mediated IBD mice after EPT administration (n = 3).

**Figure S8.** COX-2 and ASC/NLRP3 staining plots.

**Figure S9.** Cytotoxicity of EPT toward RAW264.7 cells (n = 5).

**Figure S10.** Effects of EPT toward mRNA levels for *Mfn1*, *Mfn2*, *Opa1*, *iNOS*, *COX-2*, *IL-1α*, *IL-6*, and *CCL5* in LPS-mediated RAW264.7 cells (n = 3).

**Figure S11.** TOM20 and DNA staining plot.

**Figure S12.** Quantitative data of COX-2, iNOS, p-p65/p65, Mfn1, Mfn2, Opa1, Fis1, LDHA, GLUT1, and GSDMD-N/GSDMD in LPS-mediated RAW264.7 cells after EPT administration (n = 3)

**Figure S13.** JC-1 staining plot.

**Figure S14.** Quantitative data of cleaved caspase 1/caspase 1, IL-1β, pro IL-1β, ASC, and NLRP3 in LPS plus NIG-mediated RAW264.7 cells after EPT administration (n = 3).

**Figure S15.** (A) Quantitative data of DARTS and CETSA (n = 3); (B) Immunofluorescence co-localization of EPT with PKM2.

**Figure S16.** Effects of EPT toward expressions for iNOS, Drp1, Mfn1, and NLRP3 in LPS and LPS + NIG-mediated normal and PKM2 knockdown RAW264.7 cells (n = 3).

**Figure S17.** *PKM2* knockdown abolished effects of EPT toward expression of GLUT1, LHDA, COX-2, Drp1, Mfn1, Mfn2, Opa1, IL-1β, pro IL-1β, Cleaved-caspase 1/Caspase 1, and ASC in LPS- or LPS plus NIG-mediated RAW264.7 cells.

**Figure S18.** *PKM2* overexpression weakened effects of EPT toward expressions of iNOS and COX-2 in LPS-mediated RAW264.7 cells.

**Figure S19.** Effects of EPT toward expressions for iNOS, COX-2, Drp1, Mfn1, and NLRP3 in LPS- or LPS + NIG-mediated normal and PKM2 overexpression RAW264.7 cells (n = 3).

**Figure S20.** GST Pull down experiment for the interaction of PKM2 with Uhrf1.

**Figure S21.** (A) Verification of PKM2 knockdown (n = 3); (B) Effect of EPT toward NLRP3 mRNA level in DSS-mediated WT and PKM2 knockdown mice (n = 3).

**Figure S22.** Rectal bleeding score in DSS-mediated PKM2 knockdown mice after EPT administration (n = 8).

**Figure S23.** Occuldin staining plot.

**Figure S24.** Flow cytometry results for Th17 (CD4^+^IL-17A^+^) and Treg (CD4^+^CD25^+^Foxp3^+^) cells (n = 3).

**Figure S25.** ^1^H NMR spectrum of Bio-EPT (600 MHz, DMSO-*d*_6_)

**Figure S26.** ^13^C NMR spectrum of Bio-EPT (150 MHz, DMSO-*d*_6_)
